# Arachidonic acid metabolism as a therapeutic target in AKI-to-CKD transition

**DOI:** 10.3389/fphar.2024.1365802

**Published:** 2024-03-08

**Authors:** Xiao-Jun Li, Ping Suo, Yan-Ni Wang, Liang Zou, Xiao-Li Nie, Ying-Yong Zhao, Hua Miao

**Affiliations:** ^1^ School of Pharmacy, Zhejiang Chinese Medical University, Hangzhou, Zhejiang, China; ^2^ Department of Nephrology, Integrated Hospital of Traditional Chinese Medicine, Southern Medical University, Guangzhou, Guangdong, China; ^3^ School of Food and Bioengineering, Chengdu University, Chengdu, Sichuan, China

**Keywords:** arachidonic acid, inflammation, oxidative stress, acute kidney injury, chronic kidney disease, renal cell carcinoma

## Abstract

Arachidonic acid (AA) is a main component of cell membrane lipids. AA is mainly metabolized by three enzymes: cyclooxygenase (COX), lipoxygenase (LOX) and cytochrome P450 (CYP450). Esterified AA is hydrolysed by phospholipase A_2_ into a free form that is further metabolized by COX, LOX and CYP450 to a wide range of bioactive mediators, including prostaglandins, lipoxins, thromboxanes, leukotrienes, hydroxyeicosatetraenoic acids and epoxyeicosatrienoic acids. Increased mitochondrial oxidative stress is considered to be a central mechanism in the pathophysiology of the kidney. Along with increased oxidative stress, apoptosis, inflammation and tissue fibrosis drive the progressive loss of kidney function, affecting the glomerular filtration barrier and the tubulointerstitium. Recent studies have shown that AA and its active derivative eicosanoids play important roles in the regulation of physiological kidney function and the pathogenesis of kidney disease. These factors are potentially novel biomarkers, especially in the context of their involvement in inflammatory processes and oxidative stress. In this review, we introduce the three main metabolic pathways of AA and discuss the molecular mechanisms by which these pathways affect the progression of acute kidney injury (AKI), diabetic nephropathy (DN) and renal cell carcinoma (RCC). This review may provide new therapeutic targets for the identification of AKI to CKD continuum.

## 1 Introduction

**FIGURE 1:**
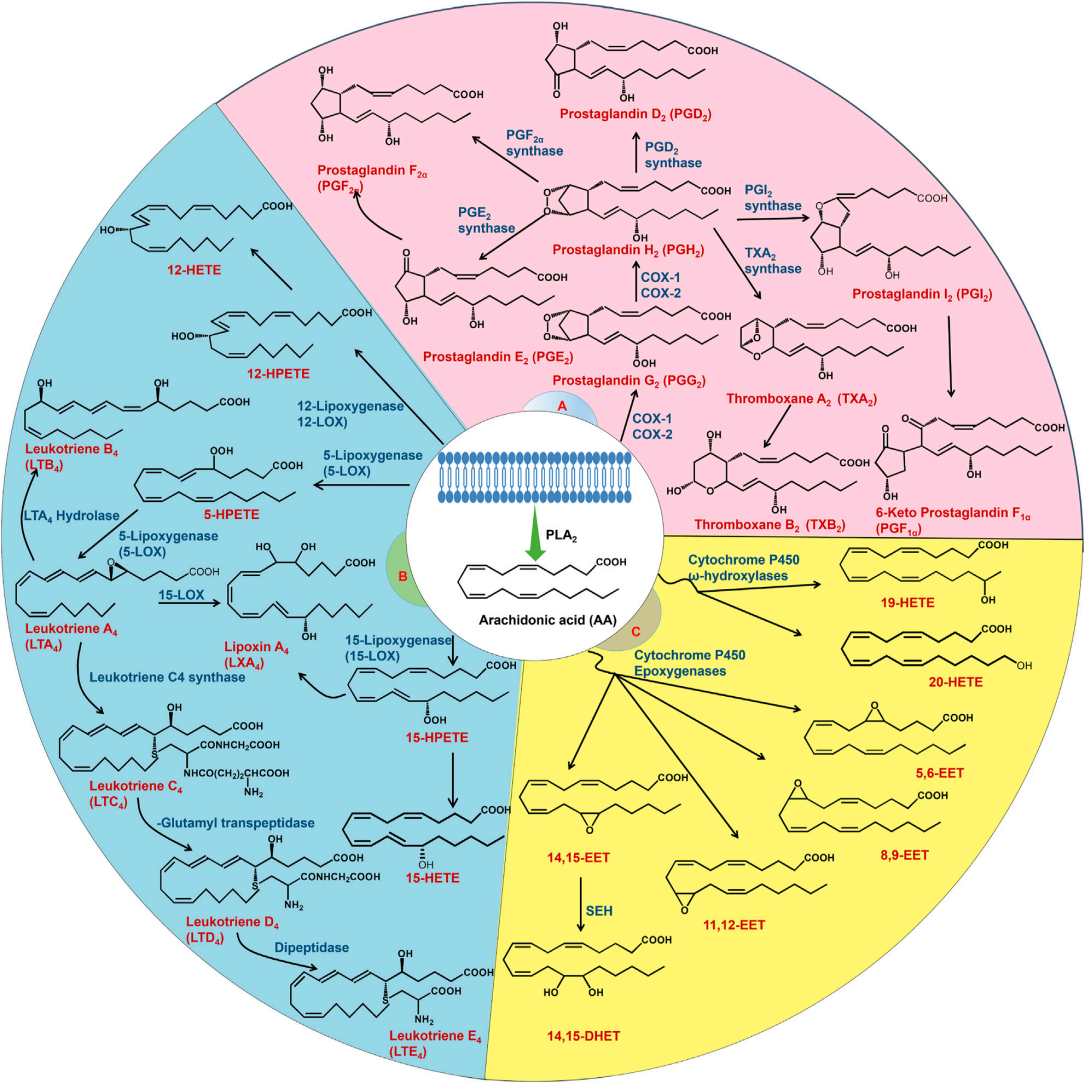
AA metabolism. AA is released from the cell membrane by PLA_2_. AA is mainly metabolized by three enzymes: COX, LOX and CYP450. The COX pathway **(A)** converts AA to prostaglandins and thromboxanes. The LOX pathway **(B)** metabolizes AA to LT, HETE and LX. AA is mainly metabolized into EET and HETE through the CYP450 pathway **(C)**. EET is converted to dihydroxyeicosatrienoic acids by sEH.

Arachidonic acid (AA) is an n-6 essential fatty acid that exists in an esterified form in the membrane phospholipids of all mammalian cells and plays an important role in human and animal growth and development (Badimon et al., 2021; Huang et al., 2021). Studies have shown that phospholipase A_2_ (PLA_2_) catalyses the hydrolysis of sn-2 acyl ester bonds in phospholipids to produce active metabolites, including lysophospholipids and AA, which alter various cell functions (Turolo et al., 2021). Free AA is further metabolized by cyclooxygenase (COX), lipoxygenase (LOX) and cytochrome P450 (CYP450) to a wide range of bioactive mediators, including prostaglandin (PG), lipoxin (LX), thromboxane (TX), leukotriene (LT), hydroxyeicosatetraenoic acid (HETE) and epoxyeicosatrienoic acid (EET) (Huang et al., 2021) (Figure 1). These AA metabolites, which are collectively known as eicosanoids, are potent autocrine and paracrine mediators with a variety of biological activities that play critical roles in normal and various pathophysiological functions (Kopp et al., 2019; Wang et al., 2023). AA and its metabolites have attracted much attention in kidney and cancer biology, especially in relation to inflammatory processes and disease (Fishbein et al., 2021). COX, LOX and CYP450 metabolites are important lipid mediators of renal function and contribute significantly to kidney dysfunction in diseases such as diabetes, hypertension, acute kidney injury (AKI) and chronic kidney disease (CKD) (Imig, 2015). Metabolic enzymes and their products in the AA pathway regulate inflammatory responses and modulate multiple cellular processes, such as angiogenesis, cell proliferation, survival, invasion and metastasis, which promote carcinogenesis (Yarla et al., 2016). COX, LOX and CYP450 and their inhibitors are widely used in the treatment of inflammation and cancer and are new targets for cancer prevention and treatment (Fishbein et al., 2021). This review will discuss the role of the AA pathway in the pathogenesis of AKI, diabetic nephropathy (DN) and renal cell carcinoma (RCC).

## 2 The subtypes and clinical features of diverse kidney diseases

Before summarizing the role of the AA signalling pathway in kidney pathogenesis, we will briefly review the subtypes and clinical features of the different kidney diseases and their different classifications. AKI is characterized by a sudden decrease in the glomerular filtration rate (GFR), which is characterized by a rapid increase in serum creatinine concentrations and/or oliguria (Ronco et al., 2019). This type of injury occurs in approximately 30%–60% of critically ill patients, and the major complications include electrolyte disorders, volume overload, uraemic complications, and drug toxicity, which are associated with acute morbidity and mortality (Pickkers et al., 2021). Even if patients with AKI survive the acute stage, AKI can develop to CKD and progress to end-stage renal disease, which causes a huge social burden (Chen et al., 2018; Chen D. Q. et al., 2019; Chen L. et al., 2019). Renal ischaemia‒reperfusion injury (IRI) has been considered an appropriate model for examining the impact of therapeutic interventions on the transition from AKI to CKD. Potential mechanisms of kidney IRI are associated with the initiation of inflammation and oxidative stress (Shuvy et al., 2011; Xiao et al., 2016).

The morbidity and mortality rates of type 1 and type 2 diabetes, both of which are costly to healthcare systems, have risen rapidly worldwide in recent decades (Zhao et al., 2019; Wu et al., 2021). DN is one of the most common microvascular complications and the leading cause of chronic and end-stage renal disease worldwide, requiring dialysis or kidney transplantation and increasing the risk of cardiovascular disease (Reidy et al., 2014; Lytvyn et al., 2020). The clinical features of DN are proteinuria and a progressive reduction in kidney function (Umanath and Lewis, 2018). Pathologically, DN is characterized by basement membrane thickening, mesangial matrix expansion, foot process effacement, nodular glomerulosclerosis and tubulointerstitial fibrosis (Anders et al., 2018). There is growing evidence that inflammation and oxidative stress are the major pathophysiological mechanisms of DN (Reidy et al., 2014; Yan, 2021).

Kidney cancer is one of the top ten cancers diagnosed in the United States in both men and women (Chen et al., 2020; Siegel et al., 2020). Kidney cancer includes a diverse spectrum of tumours. RCC is a common and fatal disease, and there are 400,000 estimated new cases and 175,000 deaths worldwide in 2018 (Linehan and Ricketts, 2019; Feng et al., 2020). RCC is the most common solid kidney tumour, originates in renal tubular epithelial cells and accounts for 87% of all renal malignancies (Bhatt and Finelli, 2014). RCC is divided into different subtypes, with clear cell RCC (ccRCC) accounting for approximately 85% of all RCC tumors, making it the most common subtype (Makhov et al., 2018). Papillary RCC and chromophobe RCC are the most common remaining histological subtypes, with incidences of 7%–14% and 6%–11%, respectively (Shuch et al., 2015). Currently, surgical resection is the first-line treatment for RCC (Wang et al., 2020). In addition, most RCCs are resistant to chemotherapy and radiotherapy once they have recurred or metastasized.

## 3 Overview of AA metabolism

AA is metabolized mainly by three enzymes: COX, LOX and CYP450. The different metabolic pathways are described separately in the following sections.

### 3.1 COX pathway

COX refers to enzymes known as prostaglandin G/H synthases (PGHS), which catalyse AA into PGH_2_, PGG_2_ and TXA. COX enzymes exist in two isoforms called COX-1 (PGHS-1) and COX-2 (PGHS-2), which are the products of two different genes (Alvarez and Lorenzetti, 2021). COX-1 is a constitutive housekeeping enzyme that is found in almost all cells and tissues, maintains baseline levels of PGs and is vital for protecting the stomach by producing mucus and maintaining renal blood flow (Burian and Gelisslinger, 2005; Hyde and Missailids, 2009). Furthermore, COX-2 is hardly or not expressed under normal physiological conditions but increases dramatically in a variety of inflammatory and cancerous states (Garavito et al., 2002). The first step in the COX metabolic pathway is the oxygenation of AA through its cyclooxygenase activity to produce PGG_2_, followed by the rapid conversion of PGG_2_ through its peroxidase activity into PGH_2_ (Hyde and Missailidis, 2009). PGH_2_ is an unstable endoperoxide that is metabolized by specific synthases to PGI_2_, PGD_2_, PGE_2_, PGF_2α_ and TXA_2_ (Honda and Kabashima, 2015). PGs exert their effects by specific membrane-localized G protein-coupled receptors on the plasma membrane, and these prostanoid receptors are classified into five basic types: the PGD receptor (DP), the PGE receptor (EP1-4), the PGF receptor (FP), the PGI receptor (IP), and the thromboxane receptor (TP) (Narumiya and Fitzgerald, 2001). PGE_2_ is the most abundant PG and has complex pathophysiological effects on the inflammatory response pathway, which is composed of four distinct and tissue-specific E prostanoid families of GPCRs: EP1-4 (Yarla et al., 2016; Wang et al., 2019). TXA synthase is a downstream enzyme of COX that catalyses the conversion of PGH_2_ to TXA_2_ (Schneider and Pozzi, 2011).

### 3.2 LOX pathway

Current research has confirmed that at least four types of enzymes in the LOX pathway (5-LOX, 8-LOX, 12-LOX, and 15-LOX) participate in the metabolism of AA (Wang et al., 2019). The four distinct LOX enzymes insert molecular oxygen at carbons 5, 8, 12, or 15 in AA, generating 5-, 8-, 12-, or 15- hydroperoxyeicosatetraenoic acid (5-, 8-, 12-, and 15-HPETE), respectively. HPETEs can be further reduced by glutathione peroxidase to HETE or converted to other biologically active compounds, such as LT and LX (Borin et al., 2017). Specifically, human 5-LOX plays a crucial role in the progression of kidney inflammation, exerting its effects across a spectrum that spans from the kidney tubules to the glomeruli (Wang et al., 2019). LTA_4_ formation is facilitated when 5-LOX interacts with the 5-LOX activating protein FLAP. FLAP is necessary for the conversion of AA to LTA_4_ because it is a membrane-spanning protein with three transmembrane domains (Rådmark et al., 2015). LTA_4_ is an unstable intermediate LT that can be converted into either LTB_4_ or cysteinyl LT (LTC_4_, LTD_4_, and LTE_4_) by specific downstream enzymes. These LTs exert physiological effects through GPCR-mediated signalling pathways (Haeggström and Funk, 2011). Regarding signalling, LTC_4_ and LTD_4_ exert their effects on vascular smooth muscle cell contraction to increase vascular permeability through CysLT1 and CysLT2 receptors. On the other hand, LTB_4_ is a potent chemotactic substance that acts via LTB_4_R (BLT1) and LTB_4_R2 (BLT2) receptors (Samuelsson et al., 1987; Hoxha, 2019). Interleukin 1β, tumour necrosis factor α, and histamine signalling have been shown to stimulate 5-LOX activity, ultimately leading to reactive oxygen species (ROS)-mediated NF-κB activation (Bonizzi et al., 1999; Anthonsen et al., 2001). In addition to 5-LOX, other LOX enzymes expressed in mammalian cells are 12-LOX, 15-LOX-1 and 15-LOX-2, which are mainly involved in the generation of HETE, LXA and LXB (Porro et al., 2014; Witola et al., 2014). The biosynthetic pathway of LX includes 5-LOX in neutrophils and 12-LOX in platelets. In neutrophils, 5-LOX generates LTA_4,_ which is then transferred to platelets, where 12-LOX generates LXA_4_ or LXB_4_ (Green et al., 2018; Zheng et al., 2020). Unlike the majority of LOX products, LXs are proresolving molecules with anti-inflammatory properties. LX binding to the G-protein coupled receptor ALX/FPR2 contributes to neutrophil migration and promotes the resolution of inflammation by delaying apoptosis in macrophages (Sodin-Semrl et al., 2004). 15-LOX-1 is encoded by the arachidonate 15-LOX gene, which metabolizes AA to LXA_4_, LXB_4_, and 15-oxo-ETE, while 15-LOX-2 metabolizes AA into 8(S)-HETE and 15-oxo-ETE (Dobrian et al., 2019; Singh and Rao, 2019).

### 3.3 CYP450 pathway

The CYP450 metabolic pathway is divided into ω-hydroxylases (CYP4 isoforms) and epoxygenases (CYP2 isoforms) (Sausville et al., 2019). Proximal straight tubules convert AA to 20-HETE and 19(S)-HETE in the kidney via ω-hydroxylase (Quigley et al., 2000). Xu et al. reported the expression of several ω-hydroxylases involved in AA metabolism in human tissues, including CYP4A11, CYP4F11, CYP4F2, CYP4F8 and CYP4F12 (Xu et al., 2013). Immunohistochemical studies have shown that the CYP4A and CYP4F isoforms are principally localized in proximal tubule cells and exhibit low immunoreactivity in collecting tubules and thick ascending limbs (Ito et al., 2006). Additionally, 20-HETE can be further oxidized to 20-carboxy-AA by ethanol dehydrogenase (Collins et al., 2005). The epoxidation of AA by CYP450 epoxygenase yields four EET system isomers: 5,6-EET, 8,9-EET, 11,12-EET and 14,15-EET. sEH hydrolyses EET to 5,6-DHET, 8,9-DHET, 11,12-DHET and 14,15-DHET, all of which are lipids with weak biological effects (Wang et al., 2019). sEH is one of the key enzymes in EET metabolism that regulates EET activity and levels *in vivo* (Wang et al., 2019). EET, an AA metabolite, is a highly polarizing factor in endothelial cells and is produced mainly in vascular endothelial cells and tissues such as the heart, muscle, kidney, pancreas, lung, gastrointestinal tract and brain (Hamzaoui et al., 2018; Pallàs et al., 2020). Thus, sEH is expressed in these organs. In most cases, a reduction in EET or increased sEH activity is associated with and contributes to kidney and cardiovascular diseases (Imig, 2018). The pattern is related to water and electrolyte homeostasis and blood pressure control (Imig et al., 2020). The physiological properties of vascular, renal, and heart EET increase blood flow, induce vasodilation, inhibit platelets, promote angiogenesis and the hyperpolarization of vascular smooth muscles, inhibit apoptosis, inhibit fibrosis and exert powerful anti-inflammatory effects (Aliwarga et al., 2018).

## 4 AA metabolites in AKI

In this section, we discuss the role of certain AA metabolites generated by COX, LOX and CYP450 in the development and progression of AKI (Table 1).

**TABLE 1:** AA metabolites and related receptors in renal diseases.

Diseases	Pathway	Product or receptor	Impact	References
AKI	COX	PGs	Counteracted the effects of vasoconstrictors, thereby helping to maintain RBF from decreasing	Yared et al. (1985)
AKI	COX	PGE_2_	Mediated oxidative stress-induced ferroptosis in renal tubular epithelial cells, exacerbating kidney damage	Liu et al. (2023)
AKI	COX	EP4/EP2	Enhanced PGE_2_-induced renal vasodilatation	Meurer et al. (2018)
AKI	COX	EP4	Modulated macrophage polarization impedes the progression of AKI to CKD	Guan et al. (2022)
AKI	LOX	12-HETE and 12/15 LOX	Stimulated inflammatory processes, oxidative stress, and apoptosis exacerbates AKI	Kar et al. (2020), Sharma et al. (2022), Mohamed and Sullivan (2023)
AKI	P450	20-HETE	Increased oxidative stress and the release of inflammatory cytokines, and intensifying IRI in renal epithelial cells	Nilakantan et al. (2008)
AKI	P450	20-HETE	Reduced cell swelling, tubular necrosis, and the physical obstruction of adjacent vasa recta capillaries, preventing a secondary decrease in medullary blood flow following IRI	Linkermann et al. (2014)
AKI	P450	14 (15)-EET	Inhibited hypoxia/reoxygenation-induced apoptosis in murine renal tubular epithelial cells	Deng et al. (2017), Hoff et al. (2019)
DN	COX	EP1	Associated with glomerular hypertrophy and proteinuria in DN, as well as the transcriptional activation of TGF-β and fibronectin	Makino et al. (2002)
DN	COX	PGE_2_-EP4	Alleviated fibrosis and markers of inflammation/fibrosis as well as apoptosis in mice	Nasrallah et al. (2016)
DN	COX	TXA-TP	Associated with the upregulation of TGF-β1, and related to markers of oxidative stress and inflammation	Xu et al. (2006), Cai et al. (2020)
DN	COX	PGI_2_	Altered matrix proteins and matrix metalloproteinases	Nasrallah and Hébert (2004)
DN	LOX	12-LOX/12(S)-HETE	Induced Ang II-related fibrogenic effects by increasing p38 MAPK and CREB transcriptional activity, and enhanced the expression of TGF-β	Guo et al. (2011), Sutariya et al. (2016), Xu et al. (2016), Dong et al. (2020)
DN	CYP450	20-HETE	Prevented Palb changes caused by focal segmental glomerulosclerosis factor and the increase in Palb induced by TGF-β1, reducing proteinuria and kidney injury	Sharma et al. (2000), Schiffer et al. (2001), Dahly-Vernon et al. (2005), Luo et al. (2009)
DN	CYP450	20-HETE	Enhanced NADPH-dependent superoxide anion production, upregulated Nox1 and Nox4 protein expression, and induced apoptosis in podocytes. Reduced inflammation, oxidative stress, and endothelial dysfunction	Eid et al. (2009)
DN	CYP450	EET	Reduced inflammation, oxidative stress, and endothelial dysfunction	Elmarakby et al. (2013), Imig, 2015; Imig and Khan (2015), Roche et al. (2015)
DN	CYP450	sEH	Impaired renal function, associated with the expression of renal inflammatory markers COX-2, vascular cell adhesion molecule-1 protein, and monocyte chemoattractant protein-1 mRNA in DN	Roche et al. (2015)
RCC	COX	COX-2-PGE_2_-EP2	Mediated the migration of Caki cells through the G protein-dependent PKA pathway	Woo et al. (2015)
RCC	COX	COX-2-PGE_2_-EP4	Promoted tumor cell migration and invasion by activating the PI3K-AKT signaling pathway	Chen et al. (2011), Wu et al. (2011), Zhang et al. (2017), Ching et al. (2020)
RCC	LOX	5/12-LOX	Crucial for the growth of RCC cells and may serve as a biomarker for RCC	Yoshimura et al. (2004a), Faronato et al. (2007)
RCC	CYP450	20-HETE	Related to the activation of MAPK, PI3K/Akt, and ROS pathways, leading to the progression of RCC.	Muthalif et al. (1998), Guo et al. (2008), Yu et al. (2011)
RCC	CYP450	EET	Activated multiple signaling pathways, including MAPK and PI3K/Akt, promoting cell proliferation, epithelial-mesenchymal transition, and anti-apoptotic processes	Zhang et al. (1997), Murai et al. (2006), Jiang et al. (2007), Nithipatikom et al. (2010)

### 4.1 Role of COX enzymes and their products in AKI

AKI is defined as an abrupt decrease in GFR leading to tubular necrosis and has a high morbidity and mortality rate (Imig, 2006; Kawakami et al., 2013). AKI is often associated with the use of β-lactam antibiotics and nonsteroidal anti-inflammatory drugs and may be mediated by allergy mechanisms (Yuan et al., 2020). COX metabolites have been implicated because COX inhibitor and NSAID treatment has been associated with AKI in patients (Imig, 2006). It has long been acknowledged that COX-derived PGs play a key role in the regulation of renal blood flow (RBF) and GFR (Walshe et al., 1984; DiBona, 1986). Under normal conditions, PGs have little effect on RBF and GFR. However, in certain pathophysiological conditions, especially in the volume contracted state, such as cirrhotic ascites and nephrotic syndrome, the maintenance of normal kidney function is dependent on PG (Huerta and Rodriguez, 2001; Hao and Breyer, 2007). Under volume-contracting conditions, substances such as catecholamines, vasopressin, and angiotensin cause constriction of the kidneys and peripheral arteries. In the kidneys, PGs act as vasodilators, countering the effects of these vasoconstrictors and thus helping to maintain RBF from falling (Yared et al., 1985). In addition, in volume-contracted conditions, COX-2 expression is dramatically increased in the macula densa and cortical thick ascending limb in humans and rodents (Kömhoff et al., 2000). PGE_2_ is the predominant and most active product of the COX-2 pathway and can maintain GFR by dilating afferent small arteries (Edwards, 1985). The receptors that mediate vasodilatory effects include cAMP-coupled prostanoid receptors: the EP2, EP4 and IP (Breyer and Breyer, 2001). EP4 and/or EP2 agonists increase the survival rate and restore lost kidney function in the mercury chloride model of AKI, supporting the protective role of EP2/4 in maintaining kidney function (Vukicevic et al., 2006). The kidney PGI_2_/TXB_2_ ratio is reduced in patients with antibiotic-induced AKI, and treatment with the PGI_2_ analogue iloprost or a TXA_2_ synthase inhibitor attenuates the decline in GFR and tubular injury that occurs during AKI (Papanikolaou et al., 1992). Interestingly, deletion of the IP receptor does not result in the same kidney damage as PGI_2_ deletion, suggesting that other signalling mechanisms may be responsible for the beneficial effects of PGI_2_ on RBF (Murata et al., 1997). Most patients with AKI associated with COX inhibitors have additional risk factors, including CKD, hypertension, congestive heart failure, chronic liver disease, and plasma volume depletion. Therefore, avoiding the use of COX-2 inhibitors in patients with AKI risk factors is vitally important to lower the incidence of COX inhibitor-associated AKI (Jia et al., 2015). However, in some specific cases, COX inhibitors have a protective effect on AKI. Feitoza et al. reported that indomethacin (a nonselective COX inhibitor) protected against ischaemia/reperfusion-induced kidney injury in rats (Feitoza et al., 2005). Selective COX-2 inhibitors can also attenuate cisplatin-induced AKI (Jia et al., 2011). Due to the complexity and diversity of AKI pathogenesis, this effect might depend on differences in pathogenic mechanisms or pathological damage in kidney disease.

### 4.2 Role of LOX enzymes and their products in AKI

It is noteworthy that while the COX pathway and its metabolites like prostaglandins have been extensively studied, the LOX pathway also plays a significant role in renal physiology and pathology. Research indicates that in gentamicin-induced AKI, ROS-mediated lipid peroxidation is significantly increased, which correlates with the enhanced expression of Arachidonate 12-lipoxygenase and production of 12-HETE (Sharma et al., 2022). Further studies suggest that sustained activation of 12/15 LOX aids in the recovery of renal damage post-ischemic injury in hypertensive rats, potentially linked to reduced inflammation and lowered levels of 12-HETE (Mohamed and Sullivan, 2023). Similarly, inhibitors of 12/15 LOX can protect against I/R-induced AKI by modulating inflammatory processes, oxidative stress, and apoptosis (Kar et al., 2020). This elaboration highlights the critical role of the LOX pathway in AKI, especially in the context of oxidative stress and inflammation, demonstrating its potential as a target for therapeutic intervention in renal diseases.

### 4.3 Role of CYP450 and their products in AKI

AA is mainly metabolized into EET and HETE through the CYP450 pathway. In this section, we discuss the role of CYP450-generated HETE and EET in AKI.

#### 4.3.1 CYP450-derived HETE in AKI

IRI is the most common cause of AKI (Muroya et al., 2015). 20-HETE has numerous effects on the regulation of kidney tubular and vascular function that have been implicated in IRI (Fan and Roman, 2017). Kidney ischaemia increases 20-HETE formation and/or release in the kidney cortex and outer medulla, but the levels of other eicosanoids are unaffected. Cortical levels of 20-HETE return to normal within 1 h of reperfusion, but kidney medullary levels remain elevated (Muroya et al., 2015). IRI is associated with vasocongestion and prolonged hypoxia in the kidney outer medulla. Intrarenal 20-HETE production is elevated after kidney ischaemia (Regner et al., 2009; Hoff et al., 2011). 20-HETE constricts the afferent arteriole and can reduce RBF and worsen IRI (Fan and Roman, 2017). 20-HETE increases oxidative stress and the release of inflammatory cytokines and potentiates IRI in kidney epithelial cells (Nilakantan et al., 2008). However, 20-HETE can also attenuate IRI. The increase in 20-HETE inhibits sodium transport in the proximal tubule and the thick ascending loop of Henle. Therefore, it may prevent the secondary decrease in medullary blood flow by reducing cell swelling, tubular necrosis and the physical occlusion of adjacent vasa recta capillaries after kidney IRI (Linkermann et al., 2014). Regner et al. reported that a 20-HETE agonist mimetic protected the secondary decrease in medullary blood flow and reduced IRI after bilateral kidney ischaemia (Regner et al., 2009). Kidney IRI is enhanced in Dahl salt-sensitive rats, which are deficient in the CYP4A protein and the production of 20-HETE. By transferring chromosome 5, which contains the CYP4A gene that is responsible for the production of 20-HETE, from Norwegian Brown rats to Dahl salt-sensitive rats, the levels of 20-HETE in the kidneys of the recipient rats were increased. This increase in 20-HETE levels provided partial protection against IRI (Muroya et al., 2015). However, another study indicated that a 20-HETE inhibitor (HET0016) or a 20-HETE antagonist (6,15–20-HEDE) protected against kidney IRI in uninephrectomized male rats (Fan et al., 2015). Further research has discovered that 20-HETE aggravates, whereas EETs ameliorate ischemia/reperfusion (I/R)-induced organ damage. However, EETs are rapidly metabolized by sEH. Therefore, in mice lacking the sEH gene, there is an increase in endogenous EET levels, but this increase leads to elevated 20-HETE levels, resulting in more severe kidney damage. This diminishes the potential beneficial effects of reduced EET degradation (Zhu et al., 2016). This indicates the complexity of the research, necessitating more comprehensive and in-depth investigations.

#### 4.3.2 CYP450-derived EET in AKI

Glycogen synthase kinase 3β (GSK3β) has been identified as a key enzyme involved in AKI. The pathogenesis of AKI is associated with a significant decrease in GSK3β phosphorylation and a significant increase in GSK3β activity (Deng et al., 2017). 14 (15)-EET significantly upregulated the expression of phosphorylated GSK3β in kidney cells and tissues and dose-dependently inhibited hypoxia/reoxygenation-induced apoptosis in murine renal tubular epithelial cells (Deng et al., 2017; Hoff et al., 2019). 14 (15)-EET also significantly attenuated the decrease in creatinine clearance induced by ischemia/reperfusion in mice (Hoff et al., 2019). Therefore, GSK3β may be an effective target for the treatment of AKI. In addition, EET analogues significantly induce GSK-3β inactivation, attenuate inflammatory cell infiltration and reduce the development of kidney tubular apoptosis (Hoff et al., 2019). The administration of EET or EET analogues is a promising therapeutic and preventive strategy for the treatment of AKI and other kidney injuries.

## 5 AA metabolites in DN

In this section, we discuss the role of some AA metabolites generated by COX, LOX and CYP450 in the development and progression of DN (Table 1). We summarize potential therapeutic targets in the pathogenesis of DN (Table 2).

**TABLE 2:** Potential therapeutic targets/strategies in the pathogenesis of DN.

Targets	Strategies	Mechanism of action	Effect on DN	References(s)
COX	COX-2 inhibition	Reduces glomerular hyperfiltration	Reduces glomerulosclerosis	Komers et al. (2001), Dey et al. (2004)
	EP1 receptor inhibition	Inhibits transcriptional activation of TGF-β and fibronectin	Reduces mesangial expansion, improves kidney and glomerular hypertrophy	Makino et al. (2002)
	TX synthase inhibition	Decreases the production of plasminogen activator inhibitor-1	Reduces the incidence of intraglomerular thrombi and glomerulosclerosis	Xu et al. (2006)
	TP receptor inhibition	Attenuates various markers of oxidative stress and inflammation	Improves histological changes	Xu et al. (2006)
LOX	LOX inhibition	Reduces p38 MAPK protein and collagen a5(IV) mRNA in podocyte	Reduces ECM expansion, glomerular hypertrophy and albuminuria	Yuan et al. (2008)
CYP450	CYP4A inhibition	Downregulates of Nox1 and Nox4 protein and mRNA expression and inhibits of NADPH oxidase activity	Attenuates albuminuria and reduces podocyte loss, apoptosis and foot process effacement	Williams et al. (2007)

### 5.1 Role of COX enzymes and their metabolites in DN

DN is characterized by glomerular hypertrophy, glomerular basement membrane thickening, expansion of the mesangium, arteriolar hyalinosis, and global glomerular sclerosis, which ultimately lead to proteinuria and kidney failure (Vivian and Rubinstein, 2002; Breyer et al., 2005). Increased GFR and ultrafiltration is a typical sign of early DN (McGowan et al., 2001). Several studies have connected diabetes with enhanced kidney COX-2 production of PG. In streptozotocin (STZ)-induced type I diabetic rats, kidney COX-2 protein expression is increased (Cheng et al., 2002). Kidney COX-2 expression is also strikingly upregulated in the thick ascending limbs and macula densa in type II diabetic models in Zucker rats and db/db mice (Komers et al., 2005; Sun et al., 2013). In diabetic patients, urinary PGE_2_ levels are increased in parallel with COX-2 activation (Jia et al., 2014). Selective COX-2 inhibition dramatically reduces glomerular hyperfiltration in STZ-induced diabetic rats, which is consistent with an increase in RBF in diabetic kidneys induced by COX-2-derived PGs (Komers et al., 2001). Refecoxib, a specific COX-2 inhibitor, reduced glomerulosclerosis and restored proteinuria to normal levels in obese Zucker rats without affecting hyperglycaemia (Dey et al., 2004). These findings strongly suggest that COX-2 may be an important therapeutic target for DN.

As mentioned previously, there is a strong interest in targeting EP receptors to better control the kidney complications of diabetes, but the properties of these homologous receptors have not been completely characterized. The role of EP receptors in DN and their potential as therapeutic targets are reviewed below. The EP1 receptor is thought to be an essential mediator of angiotensin II (Ang II)-induced hypertrophy in mesangial cells (MCs) (Qian et al., 2009). Treatment with EP1 receptor antagonists improves kidney and glomerular hypertrophy, inhibits transcriptional activation of transforming growth factor-β (TGF-β) and fibronectin, reduces mesangial expansion and prevents diabetic kidney injury in rats (Makino et al., 2002). Similarly, markers of injury and altered kidney function were attenuated in diabetic EP1-knockout mice. It has been suggested that selective inhibition of the EP1 receptor inhibits glomerular hypertrophy and proteinuria and TGF-β and fibronectin transcriptional activation, preventing the development of diabetic kidney injury in rats. PGE_2_ acts on the EP4 receptor and is closely associated with matrix turnover, the fibrotic response and apoptosis in kidney cells. Intrarenal EP4 was shown to attenuate tubulointerstitial fibrosis in unilateral ureteral obstruction mice, and EP4-knockout mice had increased fibrosis and inflammation/fibrosis markers, which was prevented by EP4 agonists (Nakagawa et al., 2012). Antagonizing the EP1 receptors or agonizing EP4 may reduce kidney injury associated with growth/apoptosis, inflammation, oxidative stress, and fibrosis and control dysfunction in diabetic patients, thereby reducing the need for kidney replacement therapies (Nasrallah et al., 2016). Furthermore, Coffman et al. showed that activation of TP by TXA_2_ increased the production of plasminogen activator inhibitor-1 and plasminogen activators by MC, which regulated glomerular thrombosis and fibrosis in inflammatory diseases. Consistent with this effect, Okumura et al. reported a reduction in intraglomerular thrombi and glomerulosclerosis following TXA_2_ inhibition in diabetic rats (Okumura et al., 2003). TP receptor antagonist treatment attenuated various markers of oxidative stress and inflammation in diabetic apolipoprotein E-deficient mice and ameliorated histological changes in DN (Xu et al., 2006). Interestingly, a general decrease in IP levels was found in cultured MCs exposed to high glucose. In addition, PGI_2_ analogues altered matrix proteins and matrix metalloproteinases, indicating that targeting IP might be a therapeutic approach to decrease matrix changes related to DN or other kidney diseases (Nasrallah and Hebert, 2004). At present, the identity of these COX-derived PGs and their receptors in the pathogenesis of DN has not been fully established, and much work is needed to clarify the exact pathways involved.

### 5.2 Role of LOX enzymes and their products in DN

There is growing evidence that LOX-derived products are involved in the pathogenesis of diabetes, including DN. High glucose levels directly increase the expression levels of 12-LOX in the MCs obtained from type I and type II diabetic mouse models (Yoshimura et al., 2004b). In rats with STZ-induced DN, podocyte-specific mRNA levels, as well as fibronectin and collagen a5(IV) protein levels, are increased, which correlate with increased 12-LOX mRNA and protein expression, increased p38 mitogen-activated protein kinase (MAPK) mRNA expression, and p38 MAPK protein activation (Ma et al., 2005; Watanabe et al., 2018). Inhibition of high glucose-induced podocyte 12-LOX activation by the 12-LOX inhibitor cinnamyl-3,4-dihydroxy-a-cyanocinnamate (CDC) decreased podocyte p38 MAPK and collagen a5(IV) mRNA and protein expression (Kang et al., 2003). An *in vitro* study demonstrated that MCs obtained from 12-LOX knockout mice grew more slowly than wild-type cells. This was associated with suppression of the responses of the downstream signalling molecules p38 MAPK, activator protein-1 and cAMP-responsive element-binding protein (CREB) (Kim et al., 2003). Thus, ample evidence suggests that activation of the 12-LOX and p38 MAPK signalling pathways can stimulate downstream immediate early genes and key signalling kinase molecules that mediate cytokine and growth factor activity and induce DN (Ma et al., 2005).

**FIGURE 2:**
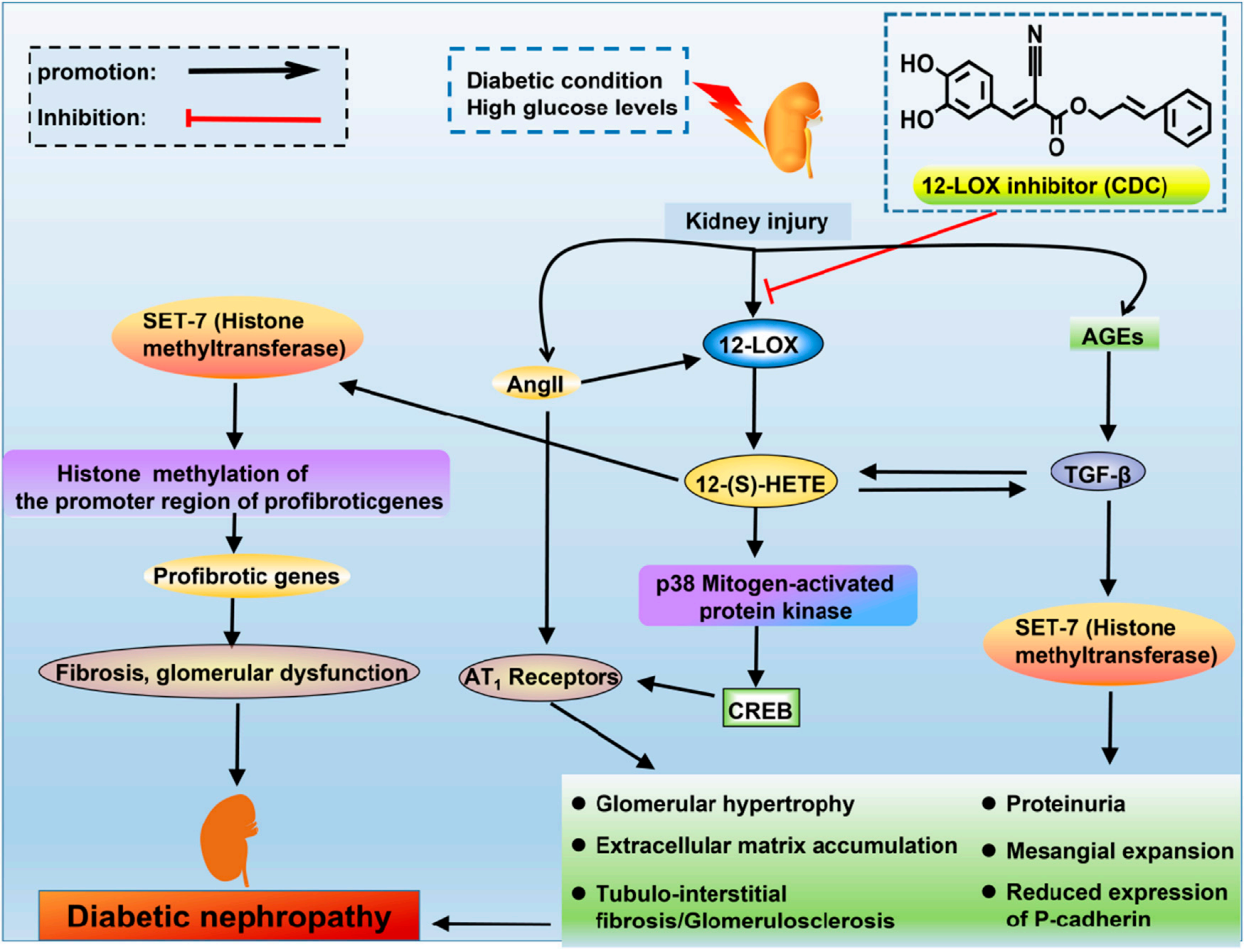
The interrelationship of TGF-β, Ang II and 12-LOX in DN. High glucose levels or diabetic conditions can activate the expression of 12-LOX, and there is a subsequent increase in the production of 12(S)-HETE. 12(S)-HETE activates p38 mitogen-activated protein kinase and CREB to increase the expression of AT1 receptors. Ang II can increase the levels of 12-LOX, which can potentiate the effects of Ang II by upregulating the expression of AT1 receptors. 12(S)-HETE can interact with TGF-β. These signals can ultimately reduce P-cadherin expression, promote mesangial expansion and induce proteinuria, which are the key events in DN. 12(S)-HETE also increases SET7 (histone methyltransferase), which can increase the expression of profibrotic genes.

Considering the key role of Ang II and TGF-β in the pathogenesis of DN, the interrelationship among Ang II, TGF-β and 12-LOX in the fibrotic changes in the diabetic kidney was investigated (Figure 2). Ang II can stimulate MC hypertrophy and ECM production, directly increasing the activity and expression of leukocyte-type 12-LOX in MCs (August and Suthanthiran, 2003). In contrast, 12-LOX-derived 12(S)-HETE induces Ang II-related fibrogenic effects by increasing p38 MAPK and CREB transcriptional activity (Dong et al., 2020). 12(S)-HETE may potentiate the effects of Ang II by upregulating the expression of AT1 receptors on the glomerulus, podocytes and the mesangium (Dong et al., 2020). The p38 MAPK inhibitor SB202190 significantly inhibits 12(S)-HETE-mediated AT1 receptor expression on rat MCs (Xu et al., 2016). These events may ultimately reduce P-cadherin expression, promote interstitial expansion, and induce proteinuria, which is a key event in DN (Guo et al., 2011). Additionally, 12(S)-HETE can cross-talk with TGF-β in an interactive manner to induce fibrotic changes in DN. It was shown that treatment of rat MCs with TGF-β increased 12(S)-HETE synthesis (Sutariya et al., 2016). Conversely, the 12-LOX product 12(S)-HETE can increase the mRNA and protein expression of TGF-β and induce TGF-β promoter activity (Kim et al., 2005). Cholesterol-tagged siRNAs targeting 12(S)-HETE ameliorated glomerular dysfunction and the expression of renal TGF-β and profibrotic genes in DN (Yuan et al., 2008). 12-LOX knockdown attenuated TGF-β-induced increases in SET7 (histone methyltransferase), histone modifications and profibrotic gene expression in mesangial cells. Transfection of MCs with siRNA targeting SET7 resulted in the silencing of SET7 and the suppression of 12(S)-HETE-induced profibrotic gene expression (Yuan et al., 2016). Thus, 12-LOX and TGF-β can interfere with and activate each other to induce the development of nephropathy in diabetic conditions.

### 5.3 Role of CYP450 and its products in DN

AA is mainly metabolized into EET and HETE through the CYP450 pathway. In this section, we discuss the role of HETE and EET produced by CYP450 in DN.

#### 5.3.1 CYP450-derived HETE in DN

Increased pressure transmission to the glomerular capillaries has an important role in the development of glomerulosclerosis. Increased glomerular capillary pressure is thought to contribute to the development of proteinuria, but the mechanisms are unclear. The increase in glomerular capillary pressure can promote the development of kidney injury by increasing TGF-β production (Williams et al., 2007). However, TGF-β has a disruptive effect on glomerular filtration, leading to glomerular damage in the early stage of DN and directly increasing the permeability of the glomerulus to albumin (Palb), which is associated with a decrease in 20-HETE formation (Sharma et al., 2000; Schiffer et al., 2001; Luo et al., 2009). 20-HETE is produced in the glomerulus and cultured podocytes. McCarthy et al. recently reported that 20-HETE has a protective effect on the glomerulus and prevents Palb changes caused by focal segmental glomerulosclerotic factor (Dahly-Vernon et al., 2005). It was found that adding a stable 20-HETE agonist (WIT003) or a 20-HETE mimetic prevented the decrease in 20-HETE levels and protected against the increase in Palb induced by TGF-β1. In addition, glomerular 20-HETE production is decreased in diabetic rats, and the induction of 20-HETE formation with fibrates decreases proteinuria and kidney injury (Fan and Roman, 2017). Overall, 20-HETE has a protective effect on the glomerular permeability barrier, in part because it can reduce transmural pressure gradients across glomerular capillaries secondary to the constriction of afferent arterioles to oppose the development of proteinuria and glomerular disease.

Oxidative stress is thought to be a key factor in the development of DN. ROS have been demonstrated to play a critical role in DN (Eid et al., 2013). However, the exact sources of ROS, as well as the molecular mechanisms of oxidative stress, have not been fully elucidated. NADPH oxidase, CYP450 and the mitochondrial electron transport chain are major sources of ROS in cells and tissues (McCarthy et al., 2015). The NADPH oxidases Nox1, Nox2 and Nox4 have recently been identified as the major sources of ROS in the glomeruli and kidney cortex in type 1 diabetic rats (Eid et al., 2009). CYP450 is a potential source of ROS in many cells and tissues (Puntarulo and Cederbsum, 1998; Fleming et al., 2001). Intracellular ROS mediate apoptosis in podocytes in response to high glucose or Ang II (Susztak et al., 2006). High glucose induces apoptosis in cultured podocytes by sequentially upregulating CYP4A, Nox1 and Nox4 to produce ROS (Bedard and Krause, 2007). 20-HETE is the major product of CYP4A and the hydroxylated product of AA, one of the major CYP450 eicosanoids produced in the kidney cortex. 20-HETE enhanced NADPH-dependent superoxide anion production, upregulated Nox1 and Nox4 protein expression, and induced apoptosis in podocytes. The type 1 diabetic mouse model OVE26 shows morphological and structural changes characteristic of human DN. Similarly, OVE26 mice exhibited glomerular basement membrane thickening, podocyte apoptosis, foot process effacement and podocyte loss (Fan et al., 2015). Injection of the specific 20-HETE production inhibitor HET0016 attenuated albuminuria and reduced podocyte loss, apoptosis and foot process effacement. CYP4A inhibition also led to the downregulation of Nox1 and Nox4 protein and mRNA expression and significant inhibition of NADPH oxidase activity (Williams et al., 2007). Collectively, these data indicate that increased release of CYP4A and 20-HETE induces podocyte injury, and this biological effect may be mediated by enhanced ROS production.

In summary, while 20-HETE exhibits a protective role in maintaining glomerular permeability and preventing proteinuria in DN, its involvement in podocyte injury through enhanced ROS production presents a complex paradox. This duality highlights the intricate balance within renal pathophysiology. Further studies are needed to unravel these conflicting roles of 20-HETE, aiming to harness its beneficial effects while mitigating the adverse outcomes related to oxidative stress and podocyte damage in diabetic nephropathy.

#### 5.3.2 CYP450-derived EET in DN

EET has been shown to be renoprotective by reducing inflammation, oxidative stress, and endothelial dysfunction in experimental animal models of diabetes and hypertension. Knockout of sEH increases the production of EET, lowers blood pressure, proteinuria, kidney inflammation and glomerular injury in animals with diabetic- and obesity-induced nephropathy and reduces renal Ang II levels and DOCA-salt-induced hypertension (Elmarakby et al., 2013; Imig, 2015; Imig and Khan, 2015; Roche et al., 2015). Recently, the production of EET was reduced in the kidney and glomeruli of STZ-treated animals, as well as in the glomeruli exposed to high glucose (Chen et al., 2012; Eid et al., 2013). Increased expression of EET or the administration of EET agonists in CYP2J2 transgenic mice or the administration of a CYP2J2 viral vector reduced proteinuria, glomerular injury and inflammation in STZ diabetic mice (Zhao et al., 2012; Hye Khan et al., 2014). In summary, the common protective effects of EET involve reducing oxidative stress and inflammation. Therefore, sEH inhibitors and EET agonists can be promising therapeutic agents for the treatment of DN.

Numerous *in vivo* and *in vitro* studies have associated the beneficial effects of EET with antiapoptotic pathways. Studies have shown that EET is not only an important regulator of apoptosis in kidney proximal tubular cells but also an effective antiapoptotic factor in the kidney (Chen et al., 2012). TNF-α-induced apoptotic HK-2 cells were treated with synthetic EET, which significantly upregulated basal antiapoptotic proteins B-cell lymphoma 2 (Bcl-2) and B-cell lymphoma-extra large (Bcl-XL) expression levels and downregulated the proapoptotic Bcl-2-associated X protein (Bax) protein expression levels (Yang et al., 2007; Hutchinson et al., 2008). Treatment of HK-2 cells with synthetic EET stimulated the phosphorylation of phosphatidylinositol 3-kinase PI3K/Akt and extracellular signal-regulated protein kinase 1/2 (ERK1/2), suggesting that kidney EET can activate the PI3K-Akt-NOS3 and AMP-activated protein kinase (AMPK) signalling pathways (Wender-Ozegowska and Biczysko, 2004; Wang et al., 2005; Xu et al., 2010). STZ-induced diabetic mice had significantly increased the levels of blood glucose, BUN, plasma creatinine, and urinary albumin and the kidney weight-to-body weight ratio. Interestingly, sEH inhibition decreased the levels of blood glucose, BUN, plasma creatinine, and urinary albumin and the kidney weight-to-body weight ratio (Chen et al., 2012). Additionally, sEH inhibition increased kidney levels of the NF-κB inhibitor IκB in the HFD mouse model of DN, resulting in decreased mRNA expression of the kidney inflammatory markers COX-2, vascular cell adhesion molecule-1 protein and monocyte chemoattractant protein-1 (Roche et al., 2015).

## 6 AA metabolites in RCC

In this section, we discuss the role of some AA metabolites generated by COX, LOX and CYP450 in the development and progression of RCC (Table 1).

### 6.1 Role of COX enzymes and their metabolites in RCC

Kidney tumour mass is sustained by the release of circulating and topically produced factors that act on cellular receptors to convert susceptible quiescent kidney cells to an activated state (Wu et al., 2011). COX-2 plays a critical pathophysiological role in the progression of RCC (Kaminska et al., 2014). Several previous studies have reported higher levels of COX-2 expression in RCC than in normal kidney (Lee et al., 2012). Currently, it is widely accepted that COX-2 is overexpressed in some human RCC cell lines and plays a key role in the carcinogenesis of human RCC by promoting PGE_2_ production and inhibiting apoptosis to subsequently enhance tumorigenesis and angiogenesis *in vivo* (Chen et al., 2004). Because of its important role in kidney tumour migration and metastasis, COX-2 is considered a promising target for cancer treatment (Woo et al., 2015). Specific COX-2 inhibitors have been used to treat cancer patients, but undesirable side effects, including cardiovascular and kidney problems, have limited their use (Ohba et al., 2011). Therefore, there is still a need for more effective and safer strategies to inhibit tumorigenesis.

COX-2 is a key enzyme in the production of PGs, and PGE_2_ is the major PG in the kidney; there is evidence of elevated PGE_2_ levels in patients diagnosed with cancer (Asano et al., 2002; Wang et al., 2006). Previous pharmacological and animal studies have reported that the primary antitumour effects of COX-2 inhibitors are mediated through the inhibition of PGE_2_. Therefore, further understanding the pathological function of PGE_2_ will be useful for future cancer treatment strategies (Hansen-Petrik et al., 2002; Zweifel et al., 2002). PGE_2_ exerts its biological effects through the G protein-coupled receptors EP1, EP2, EP3 and EP4, which can stimulate the migration of epithelial cells (Woo et al., 2015). EP2 and EP4 but not EP1 and EP3 contribute to RCC development, and different receptors mediate different signalling pathways with somewhat different outcomes. Therefore, PGE_2_ transduces multiple receptor-specific signalling events in target kidney cells.

#### 6.1.1 COX-2-PGE_2_-EP2 axis in RCC

**FIGURE 3:**
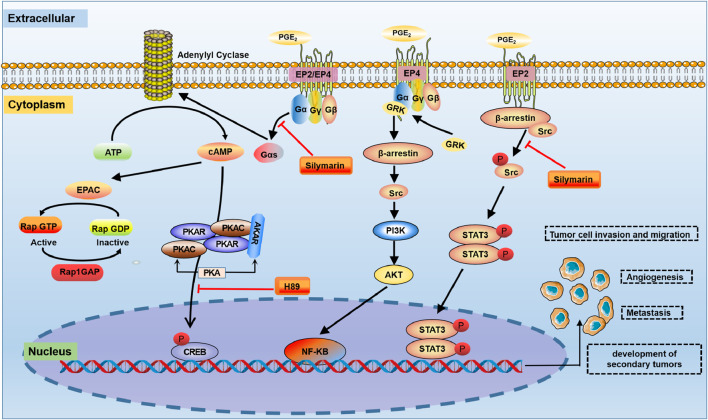
The COX-2-PGE_2_ signalling pathway in RCC. In the tumour microenvironment, PGE_2_ is involved in the regulation of a variety of signalling pathways, such as PKA, PI3K/Akt and Src-STAT3. PKA: protein kinase A; STAT3: signal transducer and activator of transcription 3.

In the Caki cell line, the PGE_2_-EP2 axis is activated the G protein-dependent PKA pathway and the G protein-independent Src-STAT3 pathway, which promotes Caki cell migration (Woo et al., 2015) (Figure 3). The PGE_2_-EP2 axis induces G protein-dependent CREB phosphorylation through the PKA signalling pathway. In this model, treatment with the PKA pathway inhibitor H89 suppressed PGE_2_-induced EP2 mRNA and protein levels, which inhibited PGE_2_-mediated migration. Similarly, a Src inhibitor (saracatinib) significantly reduced PGE_2_-mediated migration (Kaminska et al., 2014). Downregulating EP2 by knockdown and an EP2 antagonist (AH6809) reduced PGE_2_-induced Caki cell migration. In contrast, the EP2 agonist butaprost increased cell migration (Woo et al., 2015). Silymarin reduced PGE_2_-induced CREB/Src-STAT3 phosphorylation, suggesting that its inhibitory effect on PGE_2_-induced cell migration may be related to the G protein-dependent PKA-CREB and G protein-independent Src-STAT3 signalling pathways (Woo et al., 2015).

#### 6.1.2 COX-2-PGE_2_-EP4 axis in RCC

In most cell types, activated EP4 receptors are regulated by lipids and intracellular messengers such as cAMP (Fong et al., 2021) (Figure 3). The PGE_2_-EP4-mediated signalling pathway induces the dose-dependent accumulation of cAMP in RCC7 cells, which is inhibited by the ligand antagonist AH23848 (Wu et al., 2011). The actions of cAMP are mediated by PKA, ion channels and Epac. Epac is a guanine-nucleotide-exchange factor for the small GTPase Rap (Tengholm and Gylfe, 2017). There is evidence that PKA does not impact PGE_2_-regulated RCC7 cell invasion. However, Epac mediates the effects of EP4-induced RCC7 cell invasion (Wu et al., 2011). PGE_2_ induces Epac activation in RCC7 cells ectopically expressing Epac1 (Ponsioen et al., 2004). In addition, the PGE_2_-EP4 signalling pathway induced a marked increase in the level of Rap GTP-binding protein (Rap1). The accumulation of Rap1 correlated with the PGE_2_ concentration and was necessary for the increased invasion of RCC7 cells (Kim et al., 2012). Notably, the accumulation of Rap1 was inhibited when cells were treated with the EP4 antagonist AH23848. Under physiological conditions, the invasion signal is balanced by the Rap inactivator Rap1GAP, the expression of which has been lost in RCC cell lines (Zhang et al., 2017). Therefore, combined targeted inhibition of EP4 activation and the restoration of Rap1GAP expression may be a new strategy to control advanced kidney cancer.

In RCC, the EP4 receptor plays a pivotal role in cell migration and invasion. This is primarily mediated through the PI3K-AKT signaling pathway. Studies have shown that using small molecule EP4 antagonists, such as ONO-AE3-208 and AH23848, or silencing the EP4 gene, can significantly reduce the metastatic potential of RCC in preclinical models. This effect is attributed to the direct inhibition of tumor cell migration (Ching et al., 2020). Furthermore, the EP4 receptor’s involvement in the PI3K-Akt pathway extends to the activation of Ral GTPase activating protein complex 2 (RGC2) and the small GTPase RalA. These components serve as drug-targeting intermediates, offering therapeutic potential for patients with advanced RCC (Chen et al., 2011). Activation of EP4 has been observed to elevate the phosphorylation levels of Akt and RGC2, leading to increased RalA GTP levels and enhanced invasion capability of RCC cells (Zhang et al., 2017). This demonstrated a critical role of activated Akt in EP4-induced cancer cell migration and invasion (Dillon and Muller, 2010). However, the inhibition of the PI3K activation or downregulation of endogenous Akt expression results in decreased RGC2 phosphorylation. This, in turn, leads to reduced RalA GTP levels and attenuates RCC cell invasion (Li et al., 2013). These findings collectively underscore the importance of the EP4-PI3K-AKT-RGC2-RalA GTP signalling cascade in promoting RCC cell migration and invasion.

### 6.2 Role of LOX enzymes and their metabolites in RCC

There is increasing evidence that immunoreactive 5-LOX and 12-LOX are highly expressed in RCC tissues due to an alteration in normal tissue homeostasis (Yoshimura et al., 2004a; Wettersten, 2020). Transcriptional and translational levels of 5-LOX and 12-LOX were significantly upregulated in the majority of RCC compared with normal kidney tissue. Therefore, 5-LOX plays a significant role in the carcinogenesis of renal cell carcinoma and may serve as a biomarker for kidney cancer (Faronato et al., 2007). The 5-LOX protein is readily detectable in RCC cell lines Caki-1, Caki-2, and CRBM-1990. In contrast, it is undetectable in HK-2 cells derived from proximal tubules. The 12-LOX protein is significantly and strongly expressed in RCC cells A498, Caki-1, and RC-1, whereas its expression is very weak in normal kidney cells. Additionally, studies indicate that inhibitors of 5-LOX and 12-LOX have a dose- and time-dependent inhibitory effect on renal cancer cells. (Yoshimura et al., 2004a). This finding suggests that 5-LOX and 12-LOX are necessary for the growth of RCC cells. 5-LOX inhibitors may be more effective than 12-LOX inhibitors. A large amount of data suggests that 5-LOX inhibitors may be more effective than 12-LOX inhibitors in preventing the growth of cancer cells in the kidney because the specific relationship of the 5-LOX pathway is more closely related to carcinogenesis than that of the 12-LOX pathway (Matsuyama et al., 2004).

### 6.3 Role of CYP450 and its products in RCC

AA is mainly metabolized into EET and HETE through the CYP450 pathway. In this section, we discuss the role of HETE and EET produced by CYP450 in RCC.

#### 6.3.1 CYP450-derived HETE in RCC

Kidney epithelial cells typically express high levels of CYP4504A and CYP4F family enzymes and robustly produce 20-HETE, and it is likely that RCC can produce 20-HETE (Alexanian et al., 2009). The addition of HET0016 (20-HETE synthesis-selective inhibitor) and WIT002 (20-HETE antagonist) inhibited the proliferation of the human renal cell adenocarcinoma lines 786-O and 769-P but had minimal effects on the proliferation of primary human proximal tubular epithelial cells (Evangelista et al., 2020). Guo et al. showed that the CYP4/20-HETE pathway could influence tumour volume (Guo et al., 2007). The signalling mechanisms of 20-HETE-induced kidney cancer progression are mainly related to activation of the MAPK, PI3K/Akt and ROS pathways (Muthalif et al., 1998; Guo et al., 2008; Yu et al., 2011). In conclusion, targeted inhibition of 20-HETE synthesis could have renoprotective effects and reduce tumour growth in RCC.

#### 6.3.2 CYP450-derived EET in RCC

**FIGURE 4:**
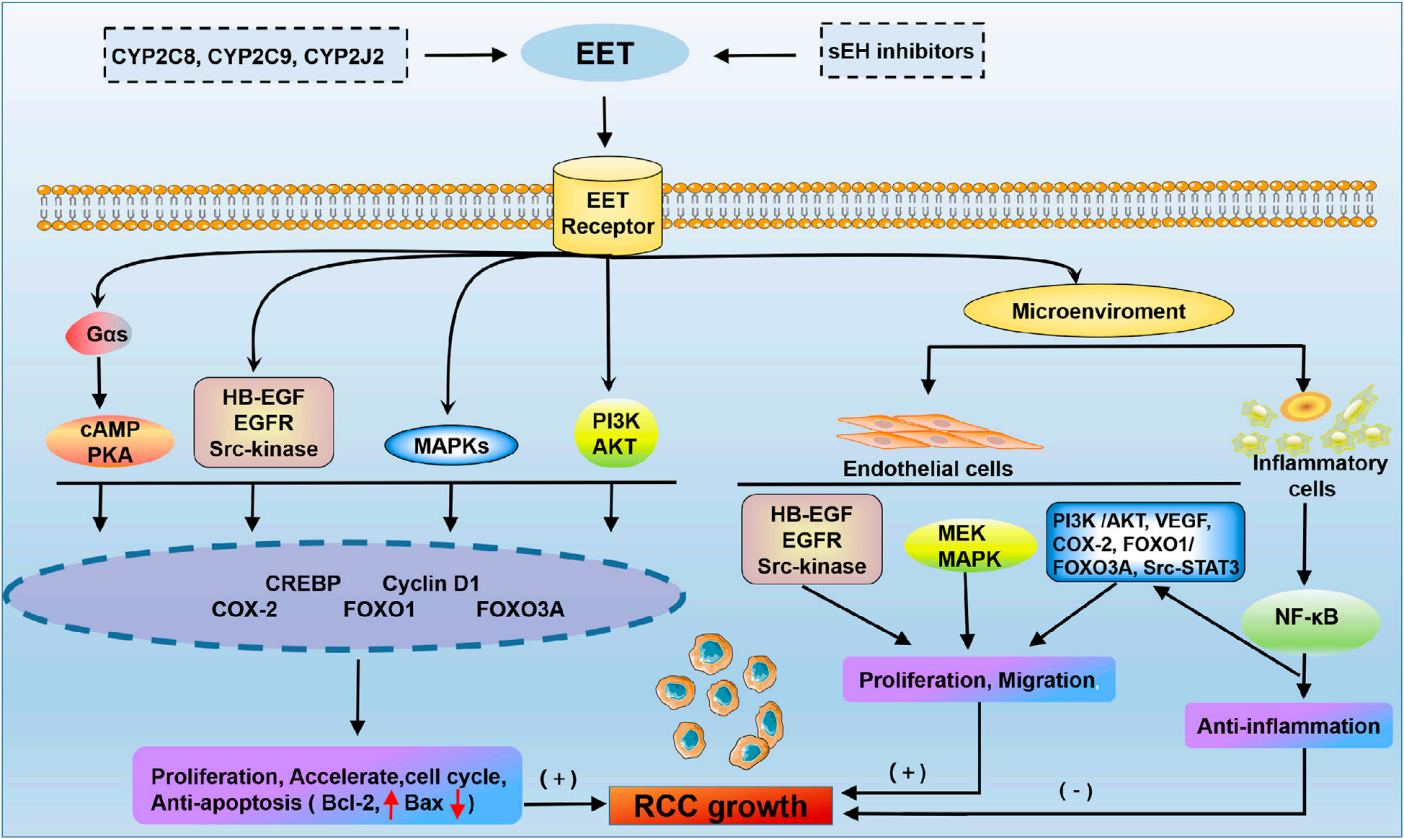
Membrane receptor mechanism of EET in RCC. The cAMP-PKA, phosphoinositide 3-kinase (PI3K)-Akt, MAPK and Src kinase pathways promote RCC growth through the activation of gene expression. EET accelerates proliferation and the cell cycle and protects carcinoma cells from apoptosis through multiple signal transduction pathways. In addition, EET has been shown to regulate multiple cells in the microenvironment by promoting endothelial cell angiogenesis and inhibiting inflammation. HB-EGF, heparin-binding epidermal growth factor-like growth factor; CREBP, cAMP response-element binding protein; VEGF, vascular endothelial growth factor.

EET plays a pivotal role in promoting the cancer phenotype of RCC (Figure 4). EET has been implicated in a wide variety of physiological processes associated with cancer pathogenesis, including the regulation of intracellular signalling pathways, cell proliferation, gene expression and inflammation (Jiang et al., 2005). The molecular mechanism of the effect of EET on cancer cell proliferation is achieved in part through significantly enhanced phosphorylation of the epidermal growth factor receptor (EGFR) and the activation of downstream signalling cascades, including the MAPK and PI3K/Akt pathways (Jiang et al., 2007; Nithipatikom et al., 2010). EET promotes epithelial-mesenchymal transition and resistance through the PI3K/Akt pathway, and this activation upregulates the metastasis-related genes MMPs and CD44. MMP activation can promote cancer cell growth and stimulate angiogenesis (Zhang et al., 1997; Murai et al., 2006). CD44 is an adhesion molecule that can activate the NF-kB signalling pathway and upregulate various prometastatic genes to further promote cancer metastasis (Fitzgerald et al., 2000). EET also inhibits apoptosis in cancer cells by upregulating the antiapoptotic proteins Bcl-2 and Bcl-XL and downregulating the proapoptotic protein Bax (Chen et al., 2009). This suggests that targeting EET signaling pathways could offer new therapeutic strategies in the treatment of RCC.

## 7 Concluding remarks

Recent advances in understanding the AA pathway highlight its significance in AKI development. COX-2 is an important physiological mediator of kidney function. COX enzymes and their products also have several beneficial renal effects by mediating processes such as RBF and renal salt handling. COX-2-derived PGs may be essential for afferent arteriolar vasodilation. During AKI, the COX product PG may act as a vasodilator to balance the constriction of renal and peripheral arteries caused by elevated levels of catecholamines, pressors and angiotensins. PG receptors can mediate vasodilatory effects and play a renoprotective role to maintain RBF. In addition, ischaemia triggers an imbalance in 20-HETE and EET in the kidney, suggesting that this imbalance plays a key role in the cascade of events that leads to renal I/R injury. 20-HETE constricts the afferent arteriole, and increased kidney 20-HETE levels can reduce RBF and worsen IRI. 20-HETE can attenuate AKI by reducing cellular swelling, tubular necrosis and the physical occlusion of adjacent vasa recta capillaries after kidney IRI. A 20-HETE agonist mimetic can protect against the secondary decrease in medullary blood flow and reduce IRI after bilateral renal ischaemia. Furthermore, the administration of EET or an EET analogue in the initiation phase of renal I/R injury may prevent ischaemic AKI.

Significant advancements have been made in understanding the AA pathway’s impact on DN. DN is associated with enhanced production of COX enzymes and the product PG. COX-2 and the product PG may be important therapeutic targets for DN. In experimental models of diabetic kidney injury, long-term treatment with selective COX-2 inhibitors and PGE_2_ receptor antagonists improves functional and structural kidney injury in these conditions. However, the identity of these COX-derived PGs and their receptors in the pathogenesis of DN has not been fully established, and much work is needed to clarify the exact pathways involved in the future. In addition, 12-LOX and its metabolite 12(S)-HETE play essential roles in the pathogenesis of DN. 12(S)-HETE may interact with angiotensin II and TGF-β to induce fibrosis in the diabetic kidney. The 12-LOX inhibitor CDC inhibited the renal 12-LOX pathway in diabetic rats *in vivo*. Therefore, the discovery of a strategy for 12-LOX inhibition with a drug regimen capable of sustained 12-LOX inhibition is important for the treatment of DN in future studies. Furthermore, EET has been shown to be renoprotective by reducing inflammation, oxidative stress, and endothelial dysfunction in experimental animal models of diabetes and hypertension. sEH inhibitors and agonists of EET can be promising therapeutic agents for the treatment of DN.

Advances in the AA pathway have shed light on its role in RCC development. COX-2 is overexpressed in some human RCC cell lines and plays a key role in the carcinogenesis of human RCC by promoting PGE_2_ production, inhibiting apoptosis and subsequently enhancing tumorigenesis and angiogenesis *in vivo*. Therefore, COX-2 may become a new target gene for RCC treatment. Specific COX-2 inhibitors have been used to treat cancer patients, but undesirable side effects, including cardiovascular and kidney problems, have limited their use. Therefore, COX downstream products and their receptors are now targets. EP2 activates the G protein-dependent PKA pathway and the G protein-independent Src-STAT3 pathway to promote RCC invasion and migration. EP4 promoted a marked increase in Akt and RGC2 phosphorylation levels, which in turn led to an increase in RalA GTP levels and increased the invasion of RCC. EP4 signalling also reduced Rap1 GAP expression and promoted RCC invasion. Therefore, the PGE_2_ receptors EP2 and EP4 could provide new therapeutic targets for RCC. Based on the reduced expression of EP4 in normal kidney tissue compared to RCC cells and the potential role of EP4 but not EP2 in malignancy aggressiveness, EP4 may be a safer and more effective target in RCC patients. In addition, the evaluation of 5-LOX and 12-LOX in preclinical studies indicates that these pathways may be more potent than COX-2, and inhibition may be a more effective therapeutic strategy (Matsuyama et al., 2004; Matsuyama et al., 2005). As a result, many new LOX inhibitors are undergoing trials for the treatment of inflammatory diseases to address the shortage of LOX inhibitors.

Many natural compounds have been shown to regulate a variety of enzymes in the AA pathway; in addition, natural compounds are readily accepted by the public and could play an important role in complementary therapies for advanced cancers.

In summary, current and ongoing studies provide directional insight into numerous unanswered questions. Future studies will gain further understanding of the role of AA metabolites in mediating oxidative stress and inflammation, as well as the overall complexity of the AA pathway and eicosanoid lipid mediators in the treatment of AKI, DKD and RCC. These findings can provide new diagnostic and prognostic methods for AKI, DKD and RCC and may establish novel therapeutic strategies for AA signalling pathway activation. Undoubtedly, future investigations will identify additional eicosanoid targets and therapies for kidney injury.

## Abbreviations

AA, Arachidonic acid; AKI, acute kidney injury; Ang II, angiotensin II; COX, cyclooxygenases; CYP450, cytochrome P450; CKD, chronic kidney disease; ccRCC, clear cell renal cell carcinoma; CREB, cAMP-responsive element-binding; DN, diabetic nephropathy; EET, epoxyeicosatrienoic acids; HETE, hydroxyeicosatetraenoic acids; IRI, ischemia-reperfusion injury; GFR, glomerular filtration rate; GSK3β, glycogen synthase kinase 3β; LOX, lipoxygenases; LX, lipoxins; LT, leukotrienes; MC, mesangial cells; PG, prostaglandins; p38, MAPK, p38 mitogen-activated protein kinase; RCC, renal cell carcinoma; ROS, reactive oxygen species; RBF, renal blood flow; STZ, streptozotocin; TGF-β, transforming growth factor-β; TX, thromboxanes.

## References

[B1] Alexanian A. Rufanova V. A. Miller B. Flasch A. Roman R. J. Sorokin A. (2009). Down-regulation of 20-HETE synthesis and signaling inhibits renal adenocarcinoma cell proliferation and tumor growth. Anticancer Res. 29, 3819–3824. 19846914 PMC2807614

[B2] Aliwarga T. Evangelista E. A. Sotoodehnia N. Lemaitre R. N. Totah R. A. (2018). Regulation of CYP2J2 and EET levels in cardiac disease and diabetes. Int. J. Mol. Sci. 19, 1916. 10.3390/ijms19071916 29966295 PMC6073148

[B3] Alvarez M. L. Lorenzetti F. (2021). Role of eicosanoids in liver repair, regeneration and cancer. Biochem. Pharmacol. 192, 114732. 10.1016/j.bcp.2021.114732 34411565

[B4] Anders H. J. Huber T. B. Isermann B. Schiffer M. (2018). CKD in diabetes: diabetic kidney disease versus nondiabetic kidney disease. Nat. Rev. Nephrol. 14, 361–377. 10.1038/s41581-018-0001-y 29654297

[B5] Anthonsen M. W. Andersen S. Solhaug A. Johansen B. (2001). Atypical λ/ι PKC conveys 5-lipoxygenase/leukotriene B4-mediated cross-talk between phospholipase A2_+_s regulating NF-κB activation in response to tumor necrosis factor-α and interleukin-1β. J. Biol. Chem. 276, 35344–35351. 10.1074/jbc.M105264200 11445585

[B6] Asano T. Shoda J. Ueda T. Kawamoto T. Todoroki T. Shimonishi M. (2002). Expressions of cyclooxygenase-2 and prostaglandin E-receptors in carcinoma of the gallbladder: crucial role of arachidonate metabolism in tumor growth and progression. Clin. Cancer Res. 8, 1157–1167. 11948128

[B7] August P. Suthanthiran M. (2003). Transforming growth factor beta and progression of renal disease. Kidney Int. Suppl, S99–S104. 10.1046/j.1523-1755.64.s87.15.x 14531781

[B8] Badimon L. Vilahur G. Rocca B. Patrono C. (2021). The key contribution of platelet and vascular arachidonic acid metabolism to the pathophysiology of atherothrombosis. Cardiovasc. Res. 117, 2001–2015. 10.1093/cvr/cvab003 33484117

[B9] Bedard K. Krause K. H. (2007). The NOX family of ROS-generating NADPH oxidases: physiology and pathophysiology. Physiol. Rev. 87, 245–313. 10.1152/physrev.00044.2005 17237347

[B10] Bhatt J. R. Finelli A. (2014). Landmarks in the diagnosis and treatment of renal cell carcinoma. Nat. Rev. Urol. 11, 517–525. 10.1038/nrurol.2014.194 25112856

[B11] Bonizzi G. Piette J. Schoonbroodt S. Greimers R. Havard L. Merville M. P. (1999). Reactive oxygen intermediate-dependent NF-κB activation by interleukin-1β requires 5-lipoxygenase or NADPH oxidase activity. Mol. Cell. Biol. 19, 1950–1960. 10.1128/mcb.19.3.1950 10022882 PMC83988

[B12] Borin T. F. Angara K. Rashid M. H. Achyut B. R. Arbab A. S. (2017). Arachidonic acid metabolite as a novel therapeutic target in breast cancer metastasis. Int. J. Mol. Sci. 18, 2661. 10.3390/ijms18122661 29292756 PMC5751263

[B13] Breyer M. D. Böttinger E. Brosius F. C. Coffman T. M. Fogo A. Harris R. C. (2005). Diabetic nephropathy: of mice and men. Adv. Chronic Kidney Dis. 12, 128–145. 10.1053/j.ackd.2005.01.004 15822049

[B14] Breyer M. D. Breyer R. M. (2001). G protein-coupled prostanoid receptors and the kidney. Annu. Rev. Physiol. 63, 579–605. 10.1146/annurev.physiol.63.1.579 11181968

[B15] Burian M. Geisslinger G. (2005). COX-dependent mechanisms involved in the antinociceptive action of NSAIDs at central and peripheral sites. Pharmacol. Ther. 107, 139–154. 10.1016/j.pharmthera.2005.02.004 15993252

[B16] Cai J. Liu B. Guo T. Zhang Y. Wu X. Leng J. (2020). Effects of thromboxane prostanoid receptor deficiency on diabetic nephropathy induced by high fat diet and streptozotocin in mice. Eur. J. Pharmacol. 882, 173254. 10.1016/j.ejphar.2020.173254 32553735

[B17] Chen C. Li G. Liao W. Wu J. Liu L. Ma D. (2009). Selective inhibitors of CYP2J2 related to terfenadine exhibit strong activity against human cancers *in vitro* and *in vivo* . J. Pharmacol. Exp. Ther. 329, 908–918. 10.1124/jpet.109.152017 19289568 PMC2683771

[B18] Chen D. Q. Feng Y. L. Chen L. Liu J. R. Wang M. Vaziri N. D. (2019a). Poricoic acid A enhances melatonin inhibition of AKI-to-CKD transition by regulating Gas6/AxlNFκB/Nrf2 axis. Free Radic. Biol. Med. 134, 484–497. 10.1016/j.freeradbiomed.2019.01.046 30716432

[B19] Chen D. Q. Hu H. H. Wang Y. N. Feng Y. L. Cao G. Zhao Y. Y. (2018). Natural products for the prevention and treatment of kidney disease. Phytomedicine 50, 50–60. 10.1016/j.phymed.2018.09.182 30466992

[B20] Chen G. Xu R. Wang Y. Wang P. Zhao G. Xu X. (2012). Genetic disruption of soluble epoxide hydrolase is protective against streptozotocin-induced diabetic nephropathy. Am. J. Physiol. Endocrinol. Metab. 303, E563–E575. 10.1152/ajpendo.00591.2011 22739108 PMC3774327

[B21] Chen L. Chen D. Q. Liu J. R. Zhang J. Vaziri N. D. Zhuang S. (2019b). Unilateral ureteral obstruction causes gut microbial dysbiosis and metabolome disorders contributing to tubulointerstitial fibrosis. Exp. Mol. Med. 51, 38. 10.1038/s12276-019-0234-2 30918245 PMC6437207

[B22] Chen Q. Shinohara N. Abe T. Watanabe T. Nonomura K. Koyanagi T. (2004). Significance of COX-2 expression in human renal cell carcinoma cell lines. Int. J. Cancer 108, 825–832. 10.1002/ijc.11646 14712483

[B23] Chen X. W. Leto D. Xiong T. Yu G. Cheng A. Decker S. (2011). A Ral GAP complex links PI3-kinase/Akt signaling to RalA activation in insulin action. Mol. Biol. Cell. 22, 141–152. 10.1091/mbc.E10-08-0665 21148297 PMC3016972

[B24] Chen Y. Y. Hu H. H. Wang Y. N. Liu J. R. Liu H. J. Liu J. L. (2020). Metabolomics in renal cell carcinoma: from biomarker identification to pathomechanism insights. Arch. Biochem. Biophys. 695, 108623. 10.1016/j.abb.2020.108623 33039388

[B25] Cheng H. F. Wang C. J. Moeckel G. W. Zhang M. Z. Mckanna J. A. Harris R. C. (2002). Cyclooxygenase-2 inhibitor blocks expression of mediators of renal injury in a model of diabetes and hypertension. Kidney Int. 62, 929–939. 10.1046/j.1523-1755.2002.00520.x 12164875

[B26] Ching M. M. Reader J. Fulton A. M. (2020). Eicosanoids in cancer: prostaglandin E_2_ receptor 4 in cancer therapeutics and immunotherapy. Front. Pharmacol. 11, 819. 10.3389/fphar.2020.00819 32547404 PMC7273839

[B27] Collins X. H. Harmon S. D. Kaduce T. L. Berst K. B. Fang X. Moore S. A. (2005). Omega-oxidation of 20-hydroxyeicosatetraenoic acid (20-HETE) in cerebral microvascular smooth muscle and endothelium by alcohol dehydrogenase 4. J. Biol. Chem. 280, 33157–33164. 10.1074/jbc.M504055200 16081420

[B28] Dahly-Vernon A. J. Sharma M. Mccarthy E. T. Savin V. J. Ledbetter S. R. Roman R. J. (2005). Transforming growth factor-β, 20-HETE interaction, and glomerular injury in Dahl salt-sensitive rats. Hypertension 45, 643–648. 10.1161/01.HYP.0000153791.89776.43 15723968

[B29] Deng B. Q. Luo Y. Kang X. Li C. B. Morisseau C. Yang J. (2017). Epoxide metabolites of arachidonate and docosahexaenoate function conversely in acute kidney injury involved in GSK3β signaling. Proc. Natl. Acad. Sci. U. S. A. 114, 12608–12613. 10.1073/pnas.1705615114 29109264 PMC5703276

[B30] Dey A. Maric C. Kaesemeyer W. H. Zaharis C. Z. Stewart J. Pollock J. S. (2004). Rofecoxib decreases renal injury in obese Zucker rats. Clin. Sci. (Lond) 107, 561–570. 10.1042/CS20040125 15307815

[B31] Dibona G. F. (1986). Prostaglandins and nonsteroidal anti-inflammatory drugs. Effects on renal hemodynamics. Am. J. Med. 80, 12–21. 10.1016/0002-9343(86)90928-9 3511683

[B32] Dillon R. L. Muller W. J. (2010). Distinct biological roles for the akt family in mammary tumor progression. Cancer Res. 70, 4260–4264. 10.1158/0008-5472.CAN-10-0266 20424120 PMC2880222

[B33] Dobrian A. D. Morris M. A. Taylor-Fishwick D. A. Holman T. R. Imai Y. Mirmira R. G. (2019). Role of the 12-lipoxygenase pathway in diabetes pathogenesis and complications. Pharmacol. Ther. 195, 100–110. 10.1016/j.pharmthera.2018.10.010 30347209 PMC6397662

[B34] Dong R. Bai M. Zhao J. Wang D. Ning X. Sun S. (2020). A comparative study of the gut microbiota associated with immunoglobulin A nephropathy and membranous nephropathy. Front. Cell. Infect. Microbiol. 10, 557368. 10.3389/fcimb.2020.557368 33194798 PMC7606180

[B35] Edwards R. M. (1985). Effects of prostaglandins on vasoconstrictor action in isolated renal arterioles. Am. J. Physiol. 248, F779–F784. 10.1152/ajprenal.1985.248.6.F779 3923841

[B36] Eid A. A. Gorin Y. Fagg B. M. Maalouf R. Barnes J. L. Block K. (2009). Mechanisms of podocyte injury in diabetes: role of cytochrome P450 and NADPH oxidases. Diabetes 58, 1201–1211. 10.2337/db08-1536 19208908 PMC2671039

[B37] Eid S. Maalouf R. Jaffa A. A. Nassif J. Hamdy A. Rashid A. (2013). 20-HETE and EETs in diabetic nephropathy: a novel mechanistic pathway. PLoS One 8, e70029. 10.1371/journal.pone.0070029 23936373 PMC3732284

[B38] Elmarakby A. A. Faulkner J. Pye C. Rouch K. Alhashim A. Maddipati K. R. (2013). Role of haem oxygenase in the renoprotective effects of soluble epoxide hydrolase inhibition in diabetic spontaneously hypertensive rats. Clin. Sci. (Lond) 125, 349–359. 10.1042/CS20130003 23611540

[B39] Evangelista E. A. Cho C. W. Aliwarga T. Totah R. A. (2020). Expression and function of eicosanoid-producing cytochrome P450 enzymes in solid tumors. Front. Pharmacol. 11, 828. 10.3389/fphar.2020.00828 32581794 PMC7295938

[B40] Fan F. Muroya Y. Roman R. J. (2015). Cytochrome P450 eicosanoids in hypertension and renal disease. Curr. Opin. Nephrol. Hypertens. 24, 37–46. 10.1097/MNH.0000000000000088 25427230 PMC4260681

[B41] Fan F. Roman R. J. (2017). Effect of cytochrome P450 metabolites of arachidonic acid in nephrology. J. Am. Soc. Nephrol. 28, 2845–2855. 10.1681/ASN.2017030252 28701518 PMC5619976

[B42] Faronato M. Muzzonigro G. Milanese G. Menna C. Bonfigli A. R. Catalano A. (2007). Increased expression of 5-lipoxygenase is common in clear cell renal cell carcinoma. Histol. Histopathol. 22, 1109–1118. 10.14670/HH-22.1109 17616938

[B43] Feitoza C. Q. Câmara N. O. Pinheiro H. S. Gonçalves G. M. Cenedeze M. A. Pacheco-Silva A. (2005). Cyclooxygenase 1 and/or 2 blockade ameliorates the renal tissue damage triggered by ischemia and reperfusion injury. Int. Immunopharmacol. 5, 79–84. 10.1016/j.intimp.2004.09.024 15589463

[B44] Feng Y. L. Chen D. Q. Vaziri N. D. Guo Y. Zhao Y. Y. (2020). Small molecule inhibitors of epithelial-mesenchymal transition for the treatment of cancer and fibrosis. Med. Res. Rev. 40, 54–78. 10.1002/med.21596 31131921

[B45] Fishbein A. Hammock B. D. Serhan C. N. Panigrahy D. (2021). Carcinogenesis: failure of resolution of inflammation? Pharmacol. Ther. 218, 107670. 10.1016/j.pharmthera.2020.107670 32891711 PMC7470770

[B46] Fitzgerald K. A. Bowie A. G. Skeffington B. S. O'neill L. A. (2000). Ras, protein kinase Cζ, and IκB kinases 1 and 2 are downstream effectors of CD44 during the activation of NF-κB by hyaluronic acid fragments in T-24 carcinoma cells. J. Immunol. 164, 2053–2063. 10.4049/jimmunol.164.4.2053 10657658

[B47] Fleming I. Michaelis U. R. Bredenkötter D. Fisslthaler B. Dehghani F. Brandes R. P. (2001). Endothelium-derived hyperpolarizing factor synthase (Cytochrome P450 2C9) is a functionally significant source of reactive oxygen species in coronary arteries. Circ. Res. 88, 44–51. 10.1161/01.res.88.1.44 11139472

[B48] Fong Z. Griffin C. S. Large R. J. Hollywood M. A. Thornbury K. D. Sergeant G. P. (2021). Regulation of P2X1 receptors by modulators of the cAMP effectors PKA and EPAC. Proc. Natl. Acad. Sci. U. S. A. 118, e2108094118. 10.1073/pnas.2108094118 34508006 PMC8449342

[B49] Garavito R. M. Malkowski M. G. Dewitt D. L. (2002). The structures of prostaglandin endoperoxide H synthases-1 and -2. Prostaglandins Other Lipid Mediat. 68-69, 129–152. 10.1016/s0090-6980(02)00026-6 12432914

[B50] Green A. R. Freedman C. Tena J. Tourdot B. E. Liu B. Holinstat M. (2018). 5S,15S-dihydroperoxyeicosatetraenoic acid (5,15-diHpETE) as a lipoxin intermediate: reactivity and kinetics with human leukocyte 5-lipoxygenase, platelet 12-lipoxygenase, and reticulocyte 15-lipoxygenase-1. Biochemistry 57, 6726–6734. 10.1021/acs.biochem.8b00889 30407793 PMC7270142

[B51] Guan X. Liu Y. Xin W. Qin S. Gong S. Xiao T. (2022). Activation of EP4 alleviates AKI-to-CKD transition through inducing CPT2-mediated lipophagy in renal macrophages. Front. Pharmacol. 13, 1030800. 10.3389/fphar.2022.1030800 36467025 PMC9709464

[B52] Guo A. M. Arbab A. S. Falck J. R. Chen P. Edwards P. A. Roman R. J. (2007). Activation of vascular endothelial growth factor through reactive oxygen species mediates 20-hydroxyeicosatetraenoic acid-induced endothelial cell proliferation. J. Pharmacol. Exp. Ther. 321, 18–27. 10.1124/jpet.106.115360 17210799

[B53] Guo A. M. Sheng J. Scicli G. M. Arbab A. S. Lehman N. L. Edwards P. A. (2008). Expression of CYP4A1 in U251 human glioma cell induces hyperproliferative phenotype *in vitro* and rapidly growing tumors *in vivo* . J. Pharmacol. Exp. Ther. 327, 10–19. 10.1124/jpet.108.140889 18591218 PMC2636507

[B54] Guo Q. Y. Miao L. N. Li B. Ma F. Z. Liu N. Cai L. (2011). Role of 12-lipoxygenase in decreasing P-cadherin and increasing angiotensin II type 1 receptor expression according to glomerular size in type 2 diabetic rats. Am. J. Physiol. Endocrinol. Metab. 300, E708–E716. 10.1152/ajpendo.00624.2010 21285403

[B55] Haeggström J. Z. Funk C. D. (2011). Lipoxygenase and leukotriene pathways: biochemistry, biology, and roles in disease. Chem. Rev. 111, 5866–5898. 10.1021/cr200246d 21936577

[B56] Hamzaoui M. Guerrot D. Djerada Z. Duflot T. Richard V. Bellien J. (2018). Cardiovascular consequences of chronic kidney disease, impact of modulation of epoxyeicosatrienoic acids. Ann. Cardiol. Angeiol. (Paris) 67, 141–148. 10.1016/j.ancard.2018.04.018 29793671

[B57] Hansen-Petrik M. B. Mcentee M. F. Jull B. Shi H. Zemel M. B. Whelan J. (2002). Prostaglandin E_2_ protects intestinal tumors from nonsteroidal anti-inflammatory drug-induced regression in Apc(Min/+) mice. Cancer Res. 62, 403–408. 11809688

[B58] Hao C. M. Breyer M. D. (2007). Physiologic and pathophysiologic roles of lipid mediators in the kidney. Kidney Int. 71, 1105–1115. 10.1038/sj.ki.5002192 17361113

[B59] Hoff U. Bubalo G. Fechner M. Blum M. Zhu Y. Pohlmann A. (2019). A synthetic epoxyeicosatrienoic acid analogue prevents the initiation of ischemic acute kidney injury. Acta Physiol. (Oxf) 227, e13297. 10.1111/apha.13297 31077555 PMC6733619

[B60] Hoff U. Lukitsch I. Chaykovska L. Ladwig M. Arnold C. Manthati V. L. (2011). Inhibition of 20-HETE synthesis and action protects the kidney from ischemia/reperfusion injury. Kidney Int. 79, 57–65. 10.1038/ki.2010.377 20962739 PMC3813968

[B61] Honda T. Kabashima K. (2015). Prostanoids in allergy. Allergol. Int. 64, 11–16. 10.1016/j.alit.2014.08.002 25572554

[B62] Hoxha M. (2019). Duchenne muscular dystrophy: focus on arachidonic acid metabolites. Biomed. Pharmacother. 110, 796–802. 10.1016/j.biopha.2018.12.034 30554118

[B63] Huang N. Wang M. Peng J. Wei H. (2021). Role of arachidonic acid-derived eicosanoids in intestinal innate immunity. Crit. Rev. Food Sci. Nutr. 61, 2399–2410. 10.1080/10408398.2020.1777932 32662287

[B64] Huerta C. Rodríguez L. A. (2001). Incidence of ocular melanoma in the general population and in glaucoma patients. J. Epidemiol. Community Health 55, 338–339. 10.1136/jech.55.5.338 11297655 PMC1731887

[B65] Hutchinson D. S. Summers R. J. Bengtsson T. (2008). Regulation of AMP-activated protein kinase activity by G-protein coupled receptors: potential utility in treatment of diabetes and heart disease. Pharmacol. Ther. 119, 291–310. 10.1016/j.pharmthera.2008.05.008 18606183

[B66] Hyde C. A. Missailidis S. (2009). Inhibition of arachidonic acid metabolism and its implication on cell proliferation and tumour-angiogenesis. Int. Immunopharmacol. 9, 701–715. 10.1016/j.intimp.2009.02.003 19239926

[B67] Hye Khan M. A. Pavlov T. S. Christain S. V. Neckář J. Staruschenko A. Gauthier K. M. (2014). Epoxyeicosatrienoic acid analogue lowers blood pressure through vasodilation and sodium channel inhibition. Clin. Sci. (Lond) 127, 463–474. 10.1042/CS20130479 24707975 PMC4167712

[B68] Imig J. D. (2006). Eicosanoids and renal vascular function in diseases. Clin. Sci. (Lond) 111, 21–34. 10.1042/CS20050251 16764555

[B69] Imig J. D. (2015). Epoxyeicosatrienoic acids, hypertension, and kidney injury. Hypertension 65, 476–482. 10.1161/HYPERTENSIONAHA.114.03585 25583156 PMC4326585

[B70] Imig J. D. (2018). Prospective for cytochrome P450 epoxygenase cardiovascular and renal therapeutics. Pharmacol. Ther. 192, 1–19. 10.1016/j.pharmthera.2018.06.015 29964123 PMC6263841

[B71] Imig J. D. Jankiewicz W. K. Khan A. H. (2020). Epoxy fatty acids: from salt regulation to kidney and cardiovascular therapeutics: 2019 Lewis K. Dahl memorial lecture. Hypertension 76, 3–15. 10.1161/HYPERTENSIONAHA.120.13898 32475311 PMC7448548

[B72] Imig J. D. Khan M. A. (2015). Cytochrome P450 and lipoxygenase metabolites on renal function. Compr. Physiol. 6, 423–441. 10.1002/cphy.c150009 26756638 PMC5965260

[B73] Ito O. Nakamura Y. Tan L. Ishizuka T. Sasaki Y. Minami N. (2006). Expression of cytochrome P-450 4 enzymes in the kidney and liver: regulation by PPAR and species-difference between rat and human. Mol. Cell. Biochem. 284, 141–148. 10.1007/s11010-005-9038-x 16552476

[B74] Jia Z. Sun Y. Liu S. Liu Y. Yang T. (2014). COX-2 but not mPGES-1 contributes to renal PGE2 induction and diabetic proteinuria in mice with type-1 diabetes. PLoS One 9, e93182. 10.1371/journal.pone.0093182 24984018 PMC4077725

[B75] Jia Z. Wang N. Aoyagi T. Wang H. Liu H. Yang T. (2011). Amelioration of cisplatin nephrotoxicity by genetic or pharmacologic blockade of prostaglandin synthesis. Kidney Int. 79, 77–88. 10.1038/ki.2010.331 20844471

[B76] Jia Z. Zhang Y. Ding G. Heiney K. M. Huang S. Zhang A. (2015). Role of COX-2/mPGES-1/prostaglandin E2 cascade in kidney injury. Mediat. Inflamm. 2015, 147894. 10.1155/2015/147894 PMC4333324 25729216

[B77] Jiang J. G. Chen C. L. Card J. W. Yang S. Chen J. X. Fu X. N. (2005). Cytochrome P450 2J2 promotes the neoplastic phenotype of carcinoma cells and is up-regulated in human tumors. Cancer Res. 65, 4707–4715. 10.1158/0008-5472.CAN-04-4173 15930289

[B78] Jiang J. G. Ning Y. G. Chen C. Ma D. Liu Z. J. Yang S. (2007). Cytochrome p450 epoxygenase promotes human cancer metastasis. Cancer Res. 67, 6665–6674. 10.1158/0008-5472.CAN-06-3643 17638876

[B79] Kaminska K. Szczylik C. Lian F. Czarnecka A. M. (2014). The role of prostaglandin E_2_ in renal cell cancer development: future implications for prognosis and therapy. Future Oncol. 10, 2177–2187. 10.2217/fon.14.152 25471032

[B80] Kang S. W. Natarajan R. Shahed A. Nast C. C. Lapage J. Mundel P. (2003). Role of 12-lipoxygenase in the stimulation of p38 mitogen-activated protein kinase and collagen α5(IV) in experimental diabetic nephropathy and in glucose-stimulated podocytes. J. Am. Soc. Nephrol. 14, 3178–3187. 10.1097/01.asn.0000099702.16315.de 14638916

[B81] Kar F. Hacioglu C. Senturk H. Donmez D. B. Kanbak G. Uslu S. (2020). Curcumin and LOXblock-1 ameliorate ischemia-reperfusion induced inflammation and acute kidney injury by suppressing the semaphorin-plexin pathway. Life Sci. 256, 118016. 10.1016/j.lfs.2020.118016 32603817

[B82] Kawakami T. Ren S. Duffield J. S. (2013). Wnt signalling in kidney diseases: dual roles in renal injury and repair. J. Pathol. 229, 221–231. 10.1002/path.4121 23097132

[B83] Kim W. J. Gersey Z. Daaka Y. (2012). Rap1GAP regulates renal cell carcinoma invasion. Cancer Lett. 320, 65–71. 10.1016/j.canlet.2012.01.022 22266190 PMC3319804

[B84] Kim Y. S. Reddy M. A. Lanting L. Adler S. G. Natarajan R. (2003). Differential behavior of mesangial cells derived from 12/15-lipoxygenase knockout mice relative to control mice. Kidney Int. 64, 1702–1714. 10.1046/j.1523-1755.2003.00286.x 14531803

[B85] Kim Y. S. Xu Z. G. Reddy M. A. Li S. L. Lanting L. Sharma K. (2005). Novel interactions between TGF-β1 actions and the 12/15-lipoxygenase pathway in mesangial cells. J. Am. Soc. Nephrol. 16, 352–362. 10.1681/ASN.2004070568 15615821

[B86] Komers R. Lindsley J. N. Oyama T. T. Schutzer W. E. Reed J. F. Mader S. L. (2001). Immunohistochemical and functional correlations of renal cyclooxygenase-2 in experimental diabetes. J. Clin. Invest. 107, 889–898. 10.1172/JCI10228 11285308 PMC199567

[B87] Komers R. Zdychová J. Cahová M. Kazdová L. Lindsley J. N. Anderson S. (2005). Renal cyclooxygenase-2 in obese Zucker (fatty) rats. Kidney Int. 67, 2151–2158. 10.1111/j.1523-1755.2005.00320.x 15882258

[B88] Kömhoff M. Jeck N. D. Seyberth H. W. Gröne H. J. Nüsing R. M. Breyer M. D. (2000). Cyclooxygenase-2 expression is associated with the renal macula densa of patients with Bartter-like syndrome. Kidney Int. 58, 2420–2424. 10.1046/j.1523-1755.2000.00425.x 11115075

[B89] Kopp B. T. Thompson R. Kim J. Konstan R. Diaz A. Smith B. (2019). Secondhand smoke alters arachidonic acid metabolism and inflammation in infants and children with cystic fibrosis. Thorax 74, 237–246. 10.1136/thoraxjnl-2018-211845 30661024 PMC7642975

[B90] Lee J. W. Park J. H. Suh J. H. Nam K. H. Choe J. Y. Jung H. Y. (2012). Cyclooxygenase-2 expression and its prognostic significance in clear cell renal cell carcinoma. Korean J. Pathol. 46, 237–245. 10.4132/KoreanJPathol.2012.46.3.237 23110009 PMC3479766

[B91] Li Z. Zhang Y. Kim W. J. Daaka Y. (2013). PGE2 promotes renal carcinoma cell invasion through activated RalA. Oncogene 32, 1408–1415. 10.1038/onc.2012.161 22580611 PMC3421051

[B92] Linehan W. M. Ricketts C. J. (2019). The Cancer Genome Atlas of renal cell carcinoma: findings and clinical implications. Nat. Rev. Urol. 16, 539–552. 10.1038/s41585-019-0211-5 31278395

[B93] Linkermann A. Chen G. Dong G. Kunzendorf U. Krautwald S. Dong Z. (2014). Regulated cell death in AKI. J. Am. Soc. Nephrol. 25, 2689–2701. 10.1681/ASN.2014030262 24925726 PMC4243360

[B94] Liu Y. Zhou L. Lv C. Liu L. Miao S. Xu Y. (2023). PGE2 pathway mediates oxidative stress-induced ferroptosis in renal tubular epithelial cells. FEBS J. 290, 533–549. 10.1111/febs.16609 36031392

[B95] Luo P. Zhou Y. Chang H. H. Zhang J. Seki T. Wang C. Y. (2009). Glomerular 20-HETE, EETs, and TGF-β1 in diabetic nephropathy. Am. J. Physiol. Renal. Physiol. 296, F556–F563. 10.1152/ajprenal.90613.2008 19129258 PMC2660192

[B96] Lytvyn Y. Bjornstad P. Van Raalte D. H. Heerspink H. L. Cherney D. Z. I. (2020). The new biology of diabetic kidney disease-mechanisms and therapeutic implications. Endocr. Rev. 41, 202–231. 10.1210/endrev/bnz010 31633153 PMC7156849

[B97] Ma J. Natarajan R. Lapage J. Lanting L. Kim N. Becerra D. (2005). 12/15-lipoxygenase inhibitors in diabetic nephropathy in the rat. Prostaglandins Leukot. Essent. Fatty Acids 72, 13–20. 10.1016/j.plefa.2004.06.004 15589395

[B98] Makhov P. Joshi S. Ghatalia P. Kutikov A. Uzzo R. G. Kolenko V. M. (2018). Resistance to systemic therapies in clear cell renal cell carcinoma: mechanisms and management strategies. Mol. Cancer Ther. 17, 1355–1364. 10.1158/1535-7163.MCT-17-1299 29967214 PMC6034114

[B99] Makino H. Tanaka I. Mukoyama M. Sugawara A. Mori K. Muro S. (2002). Prevention of diabetic nephropathy in rats by prostaglandin E receptor EP1-selective antagonist. J. Am. Soc. Nephrol. 13, 1757–1765. 10.1097/01.asn.0000019782.37851.bf 12089371

[B100] Matsuyama M. Yoshimura R. Mitsuhashi M. Tsuchida K. Takemoto Y. Kawahito Y. (2005). 5-Lipoxygenase inhibitors attenuate growth of human renal cell carcinoma and induce apoptosis through arachidonic acid pathway. Oncol. Rep. 14, 73–79. 15944770

[B101] Matsuyama M. Yoshimura R. Tsuchida K. Takemoto Y. Segawa Y. Shinnka T. (2004). Lipoxygenase inhibitors prevent urological cancer cell growth. Int. J. Mol. Med. 13, 665–668. 10.3892/ijmm.13.5.665 15067367

[B102] Mccarthy E. T. Zhou J. Eckert R. Genochio D. Sharma R. Oni O. (2015). Ethanol at low concentrations protects glomerular podocytes through alcohol dehydrogenase and 20-HETE. Prostaglandins Other Lipid Mediat. 116, 88–98. 10.1016/j.prostaglandins.2014.10.006 25447342 PMC4385495

[B103] Mcgowan T. Mccue P. Sharma K. (2001). Diabetic nephropathy. Clin. Lab. Med. 21, 111–146. 11321931

[B104] Meurer M. Ebert K. Schweda F. Höcherl K. (2018). The renal vasodilatory effect of prostaglandins is ameliorated in isolated-perfused kidneys of endotoxemic mice. Pflugers Arch. 470, 1691–1703. 10.1007/s00424-018-2183-3 30027346

[B105] Mohamed R. Sullivan J. C. (2023). Sustained activation of 12/15 lipoxygenase (12/15 LOX) contributes to impaired renal recovery post ischemic injury in male SHR compared to females. Mol. Med. 29, 163. 10.1186/s10020-023-00762-y 38049738 PMC10696802

[B106] Murai T. Miyauchi T. Yanagida T. Sako Y. (2006). Epidermal growth factor-regulated activation of Rac GTPase enhances CD44 cleavage by metalloproteinase disintegrin ADAM10. Biochem. J. 395, 65–71. 10.1042/BJ20050582 16390331 PMC1409701

[B107] Murata T. Ushikubi F. Matsuoka T. Hirata M. Yamasaki A. Sugimoto Y. (1997). Altered pain perception and inflammatory response in mice lacking prostacyclin receptor. Nature 388, 678–682. 10.1038/41780 9262402

[B108] Muroya Y. Fan F. Regner K. R. Falck J. R. Garrett M. R. Juncos L. A. (2015). Deficiency in the formation of 20-hydroxyeicosatetraenoic acid enhances renal ischemia-reperfusion injury. J. Am. Soc. Nephrol. 26, 2460–2469. 10.1681/ASN.2014090868 25644108 PMC4587700

[B109] Muthalif M. M. Benter I. F. Karzoun N. Fatima S. Harper J. Uddin M. R. (1998). 20-Hydroxyeicosatetraenoic acid mediates calcium/calmodulin-dependent protein kinase II-induced mitogen-activated protein kinase activation in vascular smooth muscle cells. Proc. Natl. Acad. Sci. U. S. A. 95, 12701–12706. 10.1073/pnas.95.21.12701 9770549 PMC22894

[B110] Nakagawa N. Yuhki K. Kawabe J. Fujino T. Takahata O. Kabara M. (2012). The intrinsic prostaglandin E2-EP4 system of the renal tubular epithelium limits the development of tubulointerstitial fibrosis in mice. Kidney Int. 82, 158–171. 10.1038/ki.2012.115 22513820

[B111] Narumiya S. Fitzgerald G. A. (2001). Genetic and pharmacological analysis of prostanoid receptor function. J. Clin. Invest. 108, 25–30. 10.1172/JCI13455 11435452 PMC209349

[B112] Nasrallah R. Hassouneh R. Hébert R. L. (2016). PGE_2_, kidney disease, and cardiovascular risk: beyond hypertension and diabetes. J. Am. Soc. Nephrol. 27, 666–676. 10.1681/ASN.2015050528 26319242 PMC4769209

[B113] Nasrallah R. Hébert R. L. (2004). Reduced IP receptors in STZ-induced diabetic rat kidneys and high-glucose-treated mesangial cells. Am. J. Physiol. Renal. Physiol. 287, F673–F681. 10.1152/ajprenal.00025.2004 15161601

[B114] Nilakantan V. Maenpaa C. Jia G. Roman R. J. Park F. (2008). 20-HETE-mediated cytotoxicity and apoptosis in ischemic kidney epithelial cells. Am. J. Physiol. Renal. Physiol. 294, F562–F570. 10.1152/ajprenal.00387.2007 18171997 PMC2633439

[B115] Nithipatikom K. Brody D. M. Tang A. T. Manthati V. L. Falck J. R. Williams C. L. (2010). Inhibition of carcinoma cell motility by epoxyeicosatrienoic acid (EET) antagonists. Cancer Sci. 101, 2629–2636. 10.1111/j.1349-7006.2010.01713.x 20804500 PMC3398840

[B116] Ohba K. Miyata Y. Watanabe S. Hayashi T. Kanetake H. Kanda S. (2011). Clinical significance and predictive value of prostaglandin E2 receptors (EPR) 1 - 4 in patients with renal cell carcinoma. Anticancer Res. 31, 597–605. 21378344

[B117] Okumura M. Imanishi M. Okamura M. Hosoi M. Okada N. Konishi Y. (2003). Role for thromboxane A2 from glomerular thrombi in nephropathy with type 2 diabetic rats. Life Sci. 72, 2695–2705. 10.1016/s0024-3205(03)00180-2 12679187

[B118] Pallàs M. Vázquez S. Sanfeliu C. Galdeano C. Griñán-Ferré C. (2020). Soluble epoxide hydrolase inhibition to face neuroinflammation in Parkinson's Disease: a new therapeutic strategy. Biomolecules 10, 703. 10.3390/biom10050703 32369955 PMC7277900

[B119] Papanikolaou N. Peros G. Morphake P. Gkikas G. Maraghianne D. Tsipas G. (1992). Does gentamicin induce acute renal failure by increasing renal TXA_2_ synthesis in rats? Prostagl. Leukot. Essent. Fat. Acids 45, 131–136. 10.1016/0952-3278(92)90229-c 1561232

[B120] Pickkers P. Darmon M. Hoste E. Joannidis M. Legrand M. Ostermann M. (2021). Acute kidney injury in the critically ill: an updated review on pathophysiology and management. Intensive Care Med. 47, 835–850. 10.1007/s00134-021-06454-7 34213593 PMC8249842

[B121] Ponsioen B. Zhao J. Riedl J. Zwartkruis F. Van Der Krogt G. Zaccolo M. (2004). Detecting cAMP-induced Epac activation by fluorescence resonance energy transfer: Epac as a novel cAMP indicator. EMBO Rep. 5, 1176–1180. 10.1038/sj.embor.7400290 15550931 PMC1299185

[B122] Porro B. Songia P. Squellerio I. Tremoli E. Cavalca V. (2014). Analysis, physiological and clinical significance of 12-HETE: a neglected platelet-derived 12-lipoxygenase product. J. Chromatogr. B Anal. Technol. Biomed. Life Sci. 964, 26–40. 10.1016/j.jchromb.2014.03.015 24685839

[B123] Puntarulo S. Cederbaum A. I. (1998). Production of reactive oxygen species by microsomes enriched in specific human cytochrome P450 enzymes. Free Radic. Biol. Med. 24, 1324–1330. 10.1016/s0891-5849(97)00463-2 9626590

[B124] Qian Q. Kassem K. M. Beierwaltes W. H. Harding P. (2009). PGE_2_ causes mesangial cell hypertrophy and decreases expression of cyclin D3. Nephron Physiol. 113, p7–p14. 10.1159/000232399 19672123

[B125] Quigley R. Baum M. Reddy K. M. Griener J. C. Falck J. R. (2000). Effects of 20-HETE and 19(S)-HETE on rabbit proximal straight tubule volume transport. Am. J. Physiol. Renal. Physiol. 278, F949–F953. 10.1152/ajprenal.2000.278.6.F949 10836982 PMC4124896

[B126] Rådmark O. Werz O. Steinhilber D. Samuelsson B. (2015). 5-Lipoxygenase, a key enzyme for leukotriene biosynthesis in health and disease. Biochim. Biophys. Acta 1851, 331–339. 10.1016/j.bbalip.2014.08.012 25152163

[B127] Regner K. R. Zuk A. Van Why S. K. Shames B. D. Ryan R. P. Falck J. R. (2009). Protective effect of 20-HETE analogues in experimental renal ischemia reperfusion injury. Kidney Int. 75, 511–517. 10.1038/ki.2008.600 19052533 PMC2643317

[B128] Reidy K. Kang H. M. Hostetter T. Susztak K. (2014). Molecular mechanisms of diabetic kidney disease. J. Clin. Invest. 124, 2333–2340. 10.1172/JCI72271 24892707 PMC4089448

[B129] Roche C. Guerrot D. Harouki N. Duflot T. Besnier M. Rémy-Jouet I. (2015). Impact of soluble epoxide hydrolase inhibition on early kidney damage in hyperglycemic overweight mice. Prostaglandins Other Lipid Mediat. 120, 148–154. 10.1016/j.prostaglandins.2015.04.011 26022136 PMC4575616

[B130] Ronco C. Bellomo R. Kellum J. A. (2019). Acute kidney injury. Lancet 394, 1949–1964. 10.1016/S0140-6736(19)32563-2 31777389

[B131] Samuelsson B. Dahlén S. E. Lindgren J. A. Rouzer C. A. Serhan C. N. (1987). Leukotrienes and lipoxins: structures, biosynthesis, and biological effects. Science 237, 1171–1176. 10.1126/science.2820055 2820055

[B132] Sausville L. N. Williams S. M. Pozzi A. (2019). Cytochrome P450 epoxygenases and cancer: a genetic and a molecular perspective. Pharmacol. Ther. 196, 183–194. 10.1016/j.pharmthera.2018.11.009 30521883

[B133] Schiffer M. Bitzer M. Roberts I. S. Kopp J. B. Ten Dijke P. Mundel P. (2001). Apoptosis in podocytes induced by TGF-β and Smad7. J. Clin. Invest. 108, 807–816. 10.1172/JCI12367 11560950 PMC200928

[B134] Schneider C. Pozzi A. (2011). Cyclooxygenases and lipoxygenases in cancer. Cancer Metastasis Rev. 30, 277–294. 10.1007/s10555-011-9310-3 22002716 PMC3798028

[B135] Sharma I. Liao Y. Zheng X. Kanwar Y. S. (2022). Modulation of gentamicin-induced acute kidney injury by myo-inositol oxygenase via the ROS/ALOX-12/12-HETE/GPR31 signaling pathway. JCI Insight 7, e155487. 10.1172/jci.insight.155487 35315361 PMC8986073

[B136] Sharma R. Khanna A. Sharma M. Savin V. J. (2000). Transforming growth factor-β1 increases albumin permeability of isolated rat glomeruli via hydroxyl radicals. Kidney Int. 58, 131–136. 10.1046/j.1523-1755.2000.00148.x 10886557

[B137] Shuch B. Amin A. Armstrong A. J. Eble J. N. Ficarra V. Lopez-Beltran A. (2015). Understanding pathologic variants of renal cell carcinoma: distilling therapeutic opportunities from biologic complexity. Eur. Urol. 67, 85–97. 10.1016/j.eururo.2014.04.029 24857407

[B138] Shuvy M. Abedat S. Beeri R. Valitsky M. Daher S. Kott-Gutkowski M. (2011). Raloxifene attenuates Gas6 and apoptosis in experimental aortic valve disease in renal failure. Am. J. Physiol. Heart Circ. Physiol. 300, H1829–H1840. 10.1152/ajpheart.00240.2010 21335463 PMC3774090

[B139] Siegel R. L. Miller K. D. Jemal A. (2020). Cancer statistics, 2020. CA Cancer J. Clin. 70, 7–30. 10.3322/caac.21590 31912902

[B140] Singh N. K. Rao G. N. (2019). Emerging role of 12/15-lipoxygenase (ALOX15) in human pathologies. Prog. Lipid Res. 73, 28–45. 10.1016/j.plipres.2018.11.001 30472260 PMC6338518

[B141] Sodin-Semrl S. Spagnolo A. Barbaro B. Varga J. Fiore S. (2004). Lipoxin A4 counteracts synergistic activation of human fibroblast-like synoviocytes. Int. J. Immunopathol. Pharmacol. 17, 15–25. 10.1177/039463200401700103 15000862

[B142] Sun Y. Jia Z. Liu G. Zhou L. Liu M. Yang B. (2013). PPARγ agonist rosiglitazone suppresses renal mPGES-1/PGE2 pathway in db/db mice. PPAR Res. 2013, 612971. 10.1155/2013/612971 24489534 PMC3892750

[B143] Susztak K. Raff A. C. Schiffer M. Böttinger E. P. (2006). Glucose-induced reactive oxygen species cause apoptosis of podocytes and podocyte depletion at the onset of diabetic nephropathy. Diabetes 55, 225–233. 10.2337/diabetes.55.01.06.db05-0894 16380497

[B144] Sutariya B. Jhonsa D. Saraf M. N. (2016). TGF-β: the connecting link between nephropathy and fibrosis. Immunopharmacol. Immunotoxicol. 38, 39–49. 10.3109/08923973.2015.1127382 26849902

[B145] Tengholm A. Gylfe E. (2017). cAMP signalling in insulin and glucagon secretion. Diabetes Obes. Metab. 19 (Suppl. 1), 42–53. 10.1111/dom.12993 28466587

[B146] Turolo S. Edefonti A. Mazzocchi A. Syren M. L. Morello W. Agostoni C. (2021). Role of arachidonic acid and its metabolites in the biological and clinical manifestations of idiopathic nephrotic syndrome. Int. J. Mol. Sci. 22, 5452. 10.3390/ijms22115452 34064238 PMC8196840

[B147] Umanath K. Lewis J. B. (2018). Update on diabetic nephropathy: core curriculum 2018. Am. J. Kidney Dis. 71, 884–895. 10.1053/j.ajkd.2017.10.026 29398179

[B148] Vivian E. M. Rubinstein G. B. (2002). Pharmacologic management of diabetic nephropathy. Clin. Ther. 24, 1741–1756. 10.1016/s0149-2918(02)80076-5 12501871

[B149] Vukicevic S. Simic P. Borovecki F. Grgurevic L. Rogic D. Orlic I. (2006). Role of EP2 and EP4 receptor-selective agonists of prostaglandin E_2_ in acute and chronic kidney failure. Kidney Int. 70, 1099–1106. 10.1038/sj.ki.5001715 16871242

[B150] Walshe J. J. Brentjens J. R. Costa G. G. Andres G. A. Venuto R. C. (1984). Abdominal pain associated with IgA nephropathy. Possible mechanism. Am. J. Med. 77, 765–767. 10.1016/0002-9343(84)90382-6 6385695

[B151] Wang D. Wang H. Brown J. Daikoku T. Ning W. Shi Q. (2006). CXCL1 induced by prostaglandin E2 promotes angiogenesis in colorectal cancer. J. Exp. Med. 203, 941–951. 10.1084/jem.20052124 16567391 PMC2118273

[B152] Wang T. Fu X. Chen Q. Patra J. K. Wang D. Wang Z. (2019). Arachidonic acid metabolism and kidney inflammation. Int. J. Mol. Sci. 20, 3683. 10.3390/ijms20153683 31357612 PMC6695795

[B153] Wang Y. Wei X. Xiao X. Hui R. Card J. W. Carey M. A. (2005). Arachidonic acid epoxygenase metabolites stimulate endothelial cell growth and angiogenesis via mitogen-activated protein kinase and phosphatidylinositol 3-kinase/Akt signaling pathways. J. Pharmacol. Exp. Ther. 314, 522–532. 10.1124/jpet.105.083477 15840765

[B154] Wang Y. Zhang Y. Wang P. Fu X. Lin W. (2020). Circular RNAs in renal cell carcinoma: implications for tumorigenesis, diagnosis, and therapy. Mol. Cancer 19, 149. 10.1186/s12943-020-01266-7 33054773 PMC7559063

[B155] Wang Y. N. Zhang Z. H. Liu H. J. Guo Z. Y. Zou L. Zhang Y. M. (2023). Integrative phosphatidylcholine metabolism through phospholipase A_2_ in rats with chronic kidney disease. Acta Pharmacol. Sin. 44, 393–405. 10.1038/s41401-022-00947-x 35922553 PMC9889763

[B156] Watanabe Y. Yamaguchi T. Ishihara N. Nakamura S. Tanaka S. Oka R. (2018). 7-Ketocholesterol induces ROS-mediated mRNA expression of 12-lipoxygenase, cyclooxygenase-2 and pro-inflammatory cytokines in human mesangial cells: potential role in diabetic nephropathy. Prostaglandins Other Lipid Mediat. 134, 16–23. 10.1016/j.prostaglandins.2017.11.002 29154978

[B157] Wender-Ozegowska E. Biczysko R. (2004). Vascular complications and their effect on fetal-maternal outcome and therapeutic approach in diabetic women. Ginekol. Pol. 75, 385–396. 15524413

[B158] Wettersten H. I. (2020). Reprogramming of metabolism in kidney cancer. Semin. Nephrol. 40, 2–13. 10.1016/j.semnephrol.2019.12.002 32130963

[B159] Williams J. M. Sharma M. Anjaiahh S. Falck J. R. Roman R. J. (2007). Role of endogenous CYP450 metabolites of arachidonic acid in maintaining the glomerular protein permeability barrier. Am. J. Physiol. Renal. Physiol. 293, F501–F505. 10.1152/ajprenal.00131.2007 17507602 PMC3146064

[B160] Witola W. H. Liu S. R. Montpetit A. Welti R. Hypolite M. Roth M. (2014). ALOX12 in human toxoplasmosis. Infect. Immun. 82, 2670–2679. 10.1128/IAI.01505-13 24686056 PMC4097613

[B161] Woo S. M. Min K. J. Chae I. G. Chun K. S. Kwon T. K. (2015). Silymarin suppresses the PGE_2_ -induced cell migration through inhibition of EP2 activation; G protein-dependent PKA-CREB and G protein-independent Src-STAT3 signal pathways. Mol. Carcinog. 54, 216–228. 10.1002/mc.22092 24127286

[B162] Wu J. Zhang Y. Frilot N. Kim J. I. Kim W. J. Daaka Y. (2011). Prostaglandin E2 regulates renal cell carcinoma invasion through the EP4 receptor-Rap GTPase signal transduction pathway. J. Biol. Chem. 286, 33954–33962. 10.1074/jbc.M110.187344 21832044 PMC3190815

[B163] Wu X. Q. Zhang D. D. Wang Y. N. Tan Y. Q. Yu X. Y. Zhao Y. Y. (2021). AGE/RAGE in diabetic kidney disease and ageing kidney. Free Radic. Biol. Med. 171, 260–271. 10.1016/j.freeradbiomed.2021.05.025 34019934

[B164] Xiao L. Zhou D. Tan R. J. Fu H. Zhou L. Hou F. F. (2016). Sustained activation of Wnt/β-catenin signaling drives AKI to CKD progression. J. Am. Soc. Nephrol. 27, 1727–1740. 10.1681/ASN.2015040449 26453613 PMC4884114

[B165] Xu H. Z. Cheng Y. L. Wang W. N. Wu H. Zhang Y. Y. Zang C. S. (2016). 12-Lipoxygenase inhibition on microalbuminuria in type-1 and type-2 diabetes is associated with changes of glomerular angiotensin II type 1 receptor related to insulin resistance. Int. J. Mol. Sci. 17, 684. 10.3390/ijms17050684 27164093 PMC4881510

[B166] Xu M. Ju W. Hao H. Wang G. Li P. (2013). Cytochrome P450 2J2: distribution, function, regulation, genetic polymorphisms and clinical significance. Drug Metab. Rev. 45, 311–352. 10.3109/03602532.2013.806537 23865864

[B167] Xu S. Jiang B. Maitland K. A. Bayat H. Gu J. Nadler J. L. (2006). The thromboxane receptor antagonist S18886 attenuates renal oxidant stress and proteinuria in diabetic apolipoprotein E-deficient mice. Diabetes 55, 110–119. 10.2337/diabetes.55.01.06.db05-0831 16380483

[B168] Xu X. Zhao C. X. Wang L. Tu L. Fang X. Zheng C. (2010). Increased CYP2J3 expression reduces insulin resistance in fructose-treated rats and db/db mice. Diabetes 59, 997–1005. 10.2337/db09-1241 20068141 PMC2844847

[B169] Yan L. J. (2021). NADH/NAD^+^ redox imbalance and diabetic kidney disease. Biomolecules 11, 730. 10.3390/biom11050730 34068842 PMC8153586

[B170] Yang S. Lin L. Chen J. X. Lee C. R. Seubert J. M. Wang Y. (2007). Cytochrome P-450 epoxygenases protect endothelial cells from apoptosis induced by tumor necrosis factor-α via MAPK and PI3K/Akt signaling pathways. Am. J. Physiol. Heart Circ. Physiol. 293, H142–H151. 10.1152/ajpheart.00783.2006 17322420 PMC2100428

[B171] Yared A. Kon V. Ichikawa I. (1985). Mechanism of preservation of glomerular perfusion and filtration during acute extracellular fluid volume depletion. Importance of intrarenal vasopressin-prostaglandin interaction for protecting kidneys from constrictor action of vasopressin. J. Clin. Invest. 75, 1477–1487. 10.1172/JCI111851 3998146 PMC425486

[B172] Yarla N. S. Bishayee A. Sethi G. Reddanna P. Kalle A. M. Dhananjaya B. L. (2016). Targeting arachidonic acid pathway by natural products for cancer prevention and therapy. Semin. Cancer Biol. 40-41, 48–81. 10.1016/j.semcancer.2016.02.001 26853158

[B173] Yoshimura R. Inoue K. Kawahito Y. Mitsuhashi M. Tsuchida K. Matsuyama M. (2004a). Expression of 12-lipoxygenase in human renal cell carcinoma and growth prevention by its inhibitor. Int. J. Mol. Med. 13, 41–46. 10.3892/ijmm.13.1.41 14654968

[B174] Yoshimura R. Matsuyama M. Mitsuhashi M. Takemoto Y. Tsuchida K. Kawahito Y. (2004b). Relationship between lipoxygenase and human testicular cancer. Int. J. Mol. Med. 13, 389–393. 10.3892/ijmm.13.3.389 14767568

[B175] Yu W. Chen L. Yang Y. Q. Falck J. R. Guo A. M. Li Y. (2011). Cytochrome P450 ω-hydroxylase promotes angiogenesis and metastasis by upregulation of VEGF and MMP-9 in non-small cell lung cancer. Cancer Chemother. Pharmacol. 68, 619–629. 10.1007/s00280-010-1521-8 21120482 PMC3839420

[B176] Yuan C. Ni L. Zhang C. Wu X. (2020). The role of Notch3 signaling in kidney disease. Oxid. Med. Cell. Longev. 2020, 1809408. 10.1155/2020/1809408 33149805 PMC7603621

[B177] Yuan H. Lanting L. Xu Z. G. Li S. L. Swiderski P. Putta S. (2008). Effects of cholesterol-tagged small interfering RNAs targeting 12/15-lipoxygenase on parameters of diabetic nephropathy in a mouse model of type 1 diabetes. Am. J. Physiol. Renal. Physiol. 295, F605–F617. 10.1152/ajprenal.90268.2008 18562637 PMC2519183

[B178] Yuan H. Reddy M. A. Deshpande S. Jia Y. Park J. T. Lanting L. L. (2016). Epigenetic histone modifications involved in profibrotic gene regulation by 12/15-lipoxygenase and its oxidized lipid products in diabetic nephropathy. Antioxid. Redox Signal. 24, 361–375. 10.1089/ars.2015.6372 26492974 PMC4779982

[B179] Zhang M. Wang M. H. Singh R. K. Wells A. Siegal G. P. (1997). Epidermal growth factor induces CD44 gene expression through a novel regulatory element in mouse fibroblasts. J. Biol. Chem. 272, 14139–14146. 10.1074/jbc.272.22.14139 9162042

[B180] Zhang Y. Thayele Purayil H. Black J. B. Fetto F. Lynch L. D. Masannat J. N. (2017). Prostaglandin E2 receptor 4 mediates renal cell carcinoma intravasation and metastasis. Cancer Lett. 391, 50–58. 10.1016/j.canlet.2017.01.007 28104442 PMC5340307

[B181] Zhao G. Tu L. Li X. Yang S. Chen C. Xu X. (2012). Delivery of AAV2-CYP2J2 protects remnant kidney in the 5/6-nephrectomized rat via inhibition of apoptosis and fibrosis. Hum. Gene Ther. 23, 688–699. 10.1089/hum.2011.135 22260463

[B182] Zhao H. Chen L. Yang T. Feng Y. L. Vaziri N. D. Liu B. L. (2019). Aryl hydrocarbon receptor activation mediates kidney disease and renal cell carcinoma. J. Transl. Med. 17, 302. 10.1186/s12967-019-2054-5 31488157 PMC6727512

[B183] Zheng Z. Li Y. Jin G. Huang T. Zou M. Duan S. (2020). The biological role of arachidonic acid 12-lipoxygenase (ALOX12) in various human diseases. Biomed. Pharmacother. 129, 110354. 10.1016/j.biopha.2020.110354 32540644

[B184] Zhu Y. Blum M. Hoff U. Wesser T. Fechner M. Westphal C. (2016). Renal ischemia/reperfusion injury in soluble epoxide hydrolase-deficient mice. PLoS One 11, e0145645. 10.1371/journal.pone.0145645 26727266 PMC4699807

[B185] Zweifel B. S. Davis T. W. Ornberg R. L. Masferrer J. L. (2002). Direct evidence for a role of cyclooxygenase 2-derived prostaglandin E2 in human head and neck xenograft tumors. Cancer Res. 62, 6706–6711. 12438270

